# A highly selective fluorogenic probe for the detection and *in vivo* imaging of Cu/Zn superoxide dismutase†
†Electronic supplementary information (ESI) available: Structures and characterisation for all **MK** compounds. Full characterisation data (NMR, HR-MS) for all **SODO** derivatives. See DOI: 10.1039/c6cc00095a



**DOI:** 10.1039/c6cc00095a

**Published:** 2016-03-04

**Authors:** Liyun Zhang, Jun Cheng Er, Hao Jiang, Xin Li, Zhaofeng Luo, Thomas Ramezani, Yi Feng, Mui Kee Tang, Young-Tae Chang, Marc Vendrell

**Affiliations:** a Institute of Technical Biology and Agriculture Engineering , Key Laboratory of Ion Beam Bioengineering , Hefei Institutes of Physical Science , Chinese Academy of Sciences , Hefei , Anhui 230031 , P. R. China . Email: zly0605@ustc.edu.cn; b Department of Chemistry , National University of Singapore , 3 Science Drive 2 , 117543 , Singapore; c Graduate School for Integrative Sciences and Engineering , National University of Singapore , Centre for Life Sciences , #05-01, 28 Medical Drive , 117456 Singapore; d School of Life Sciences , University of Science and Technology of China , Hefei , Anhui 230027 , P. R. China; e MRC Centre for Inflammation Research , Queen's Medical Research Institute , University of Edinburgh , EH16 4TJ Edinburgh , UK . Email: mvendrel@staffmail.ed.ac.uk

## Abstract

Copper/zinc superoxide dismutase (Cu/Zn SOD) is an essential enzyme that protects tissue from oxidative damage. Herein we report the first fluorogenic probe (SODO) for the detection and *in vivo* imaging of Cu/Zn SOD. SODO represents a unique chemical probe for translational imaging studies of Cu/Zn SOD in inflammatory disorders.

## Abstract 2

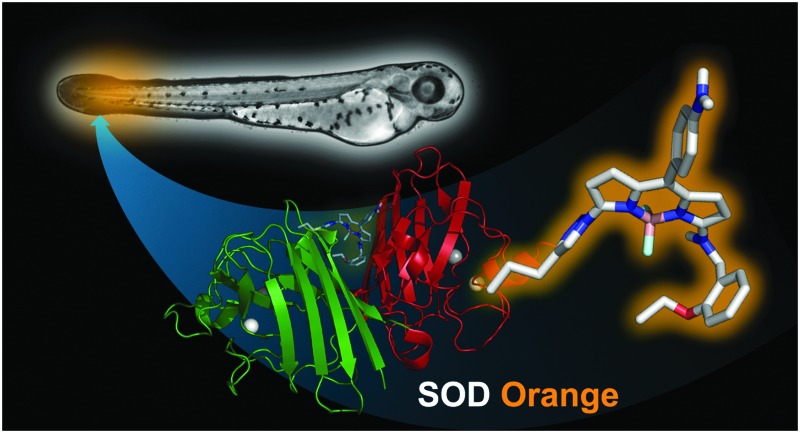
Fine-tuning the BODIPY chemical structure to develop a highly selective fluorophore for Cu/Zn SOD.

Superoxide dismutases (SOD, EC 1.15.1.1) are metalloenzymes that protect tissue from the oxidative stress caused by reactive oxygen species (ROS).^1^ The main function of SODs is to catalyse the dismutation of superoxide radicals (O_2_˙^–^) to hydrogen peroxide (H_2_O_2_) and oxygen. There are several isoforms of SODs, which can be distinguished by their metal cofactors and their distribution in cells.^2^ Among the different isoforms of SODs, copper/zinc superoxide dismutase (Cu/Zn SOD or SOD1) is widely distributed and comprises around 90% of the total SODs. Alterations in the expression and activity of Cu/Zn SOD have been associated with the onset of a number of diseases. Mutations in human Cu/Zn SOD are implicated in the development of neurological disorders, such as familial amyotrophic lateral sclerosis (fALS), Alzheimer's disease and Parkinson's disease.^3–5^ Furthermore, elevated activities of Cu/Zn SOD have been reported in cancer (*e.g.* acute myelogenous leukaemia, Hodgkin's lymphoma) and chronic inflammatory diseases (*e.g.* rheumatoid arthritis, ischemic injury).^5–7^ On the contrary, decreased levels of Cu/Zn SOD have been associated with an inhibition of the immune response and the promotion of oxidative stress in age-related disorders.^8,9^

Despite the importance of Cu/Zn SOD in regulating the balance between healthy and disease states, the exact mechanism that correlates Cu/Zn SOD to the progression of different pathologies remains largely unknown.^5^ Current probes to visualize SODs mainly rely on the intrinsic fluorescence of Tyr or Trp residues^10,11^ or the use of non-specific metal chelators, such as bathocuproine.^12^ These methods have very limited practical use *in vivo*, due to spectral shortcomings (*e.g.* short excitation/emission wavelengths) and their poor selectivity between SODs and other ROS-related enzymes.

Fluorogenic probes are advantageous for *in vivo* imaging since they provide high signal-to-noise ratios without the need for washing steps.^13,14^ Our group and others have reported the preparation of fluorogenic probes based on the 4,4-difluoro-4-bora-3*a*,4*a*-diaza-*s*-indacene (BODIPY) scaffold,^15,16^ one of the most exploited fluorophores for cell imaging due to its photostability and permeability properties.^17,18^ BODIPY fluorogens can be synthesized by direct conjugation of electron-rich groups (*e.g.* substituted benzene rings) to the BODIPY core, leading to photoinduced electron transfer (PeT) quenching and subsequent turn-on fluorescence emission in hydrophobic environments. In order to enhance the fluorogenic response of probes binding to Cu/Zn SOD, we designed a new class of BODIPY fluorogens combining PeT-quenching substituents and chemical groups restricting the rotational flexibility of the BODIPY core. The restriction of torsional motion has proven an effective strategy to generate turn-on fluorescent probes,^19,20^ and previous studies have shown that “–NH” groups directly linked to the position C_3_ of BODIPY can form intramolecular hydrogen bonds with the fluorine atoms.^21^ We synthesized **MK** fluorogens by modifying a 3,5-dichloro-BODIPY scaffold (**1**) with benzylamines (**M**) forming intramolecular hydrogen bonds with the fluorine atoms, and triazole groups (**K**) as PeT-quenchers (Scheme 1). **MK** fluorogens were prepared by loading **1** onto 2-chlorotrityl chloride polystyrene (CTC-PS) resin, followed by nucleophilic substitution and copper-catalysed azide–alkyne cycloaddition. A total of 40 **MK** compounds with diverse amine and alkyne groups were isolated in moderate to high yields with very high purities using mild acidic cleavage conditions^22^ (for detailed chemical structures and characterisation data, see ESI†).

**Scheme 1:**
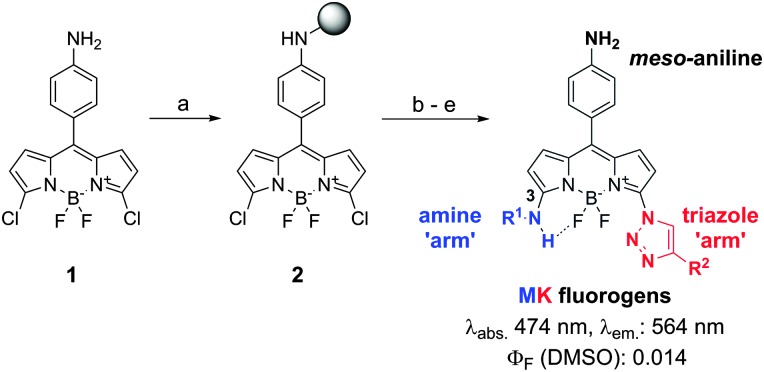
Solid-phase synthesis of **MK** fluorogens. Reaction conditions: (a) CTC-PS, DIPEA, CH_2_Cl_2_ : DMF (1 : 1), r.t.; (b) NaN_3_, DMF, r.t.; (c) R^1^NH_2_, DIPEA : DMF (1 : 4), r.t.; (d) R^2^–C≡CH, CuI, l-ascorbic acid, r.t.; (e) TFA : CH_2_Cl_2_ (0.5 : 99.5), r.t.

The spectral characterisation of the **MK** derivatives confirmed their strong fluorogenic behaviour with minimal fluorescence in aqueous media, long Stokes shifts (*i.e.* around 90 nm) and red-shifted emission wavelengths when compared to the BODIPY core (Table S1 in ESI†). As expected, the incorporation of benzylamines at the position C_3_ of the BODIPY scaffold restricted the torsional motion of the fluorophore, leading to an increase in the quantum yields in non-polar solvents. We also observed that the fluorescence emission of **MK** fluorogens correlated with solvent viscosity as a result of the decreased rotation of both triazole and aniline substituents (Fig. S1 in ESI†). Moreover, the fluorogenic response of **MK** derivatives was stronger in non-polar solvents due to the reduced PeT quenching effect from the *meso*-aniline group in non-polar environments (Fig. S2, S3 and Table S2 in ESI†). Altogether, these results assert **MK** derivatives as BODIPY fluorogens with excellent spectral properties to detect polarity changes associated to the binding at large macromolecules.

In view of these properties, we assessed our **MK** fluorogens *in vitro* to bind at the macromolecular dimeric structure of Cu/Zn SOD. The screening of diversity-oriented fluorescence libraries has become an effective strategy to identify highly selective molecular probes,^23^ and we observed that compounds with 2-ethoxybenzylamine (M103) as the amine group displayed high fluorescence emission after incubation with human Cu/Zn SOD (hCu/Zn SOD) (Fig. S4 in ESI†). **MK103-48** showed the strongest response among all compounds and was selected for further studies (hereinafter named as **SODO** (**SOD O**range), Fig. 1). **SODO** displayed up to 150-fold increase in fluorescence emission after binding at hCu/Zn SOD, with a limit of detection of 10 μg mL^–1^ (Fig. S5 in ESI†). Notably, **SODO** reached quantum yields around 45% and displayed a remarkable 60 nm hypsochromic shift in the fluorescence spectrum after binding (Fig. 1 and Fig. S5, S6 in ESI†).

**Fig. 1:**
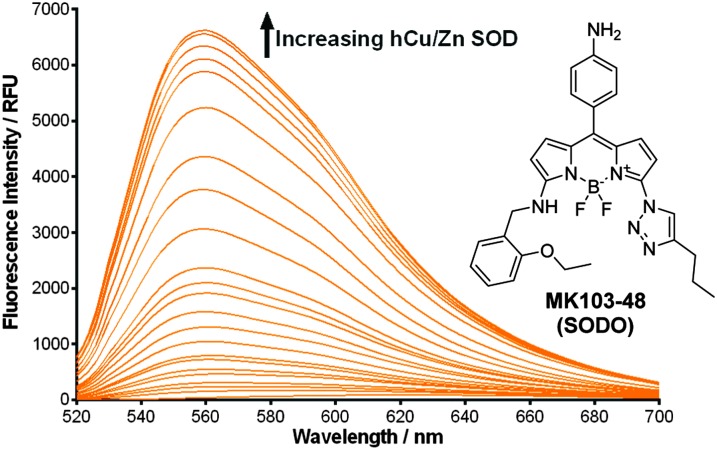
Chemical structure and fluorescence spectra of **SODO** (10 μM) after incubation with serial concentrations of hCu/Zn-SOD from 0.01 to 5 mg mL^–1^ in 20 mM Tris-HCl buffer (pH = 7.4). *λ*_exc._: 460 nm. *Φ*_F_ in hCu/Zn-SOD: 0.45.

We examined the binding of **SODO** at Cu/Zn SODs from different species. As with hCu/Zn-SOD, **SODO** displayed a concentration-dependent response in Cu/Zn SODs from bovine blood (bCu/Zn SOD) and from *Arabidopsis thaliana* (aCu/Zn SOD) (Fig. 2). These results suggest that the binding of **SODO** is species-independent and occurs at a conserved hydrophobic region of Cu/Zn SOD. While Cu/Zn SOD stands for the majority of SOD in tissue, high selectivity for the Cu/Zn SOD isoform is essential for imaging studies. We assessed the fluorescence response of **SODO** in the other two SOD isoforms (*i.e.* Mn-SOD and Fe-SOD) and observed high selectivity for Cu/Zn SOD over Mn-SOD and Fe-SOD, where minimal binding was detected (Fig. 2). To the best of our knowledge, **SODO** is the first small fluorophore able to detect Cu/Zn SOD with high specificity over other SODs. We also assessed the fluorescence emission of **SODO** in other ROS-related enzymes (*e.g.* catalase, peroxidase) (Fig. 2) and metabolites (*e.g.* H_2_O_2_, O_2_˙^–^, ^1^O_2_, OH˙) (Fig. 2, inset). **SODO** exhibited very high specificity for Cu/Zn-SOD, showing minimal fluorescence in other enzymes and metabolites involved in oxidative damage and inflammatory processes. Finally we studied whether the binding of **SODO** affected the catalytic activity of Cu/Zn SOD (Fig. S7 in ESI†). **SODO** did not significantly perturb enzymatic function; hence being an excellent reporter of Cu/Zn SOD without altering the normal physiology of cells. Altogether, these results confirm **SODO** as the first fluorogenic probe to detect Cu/Zn SOD without cross-reacting with other SOD isoforms, enzymes or ROS.

**Fig. 2:**
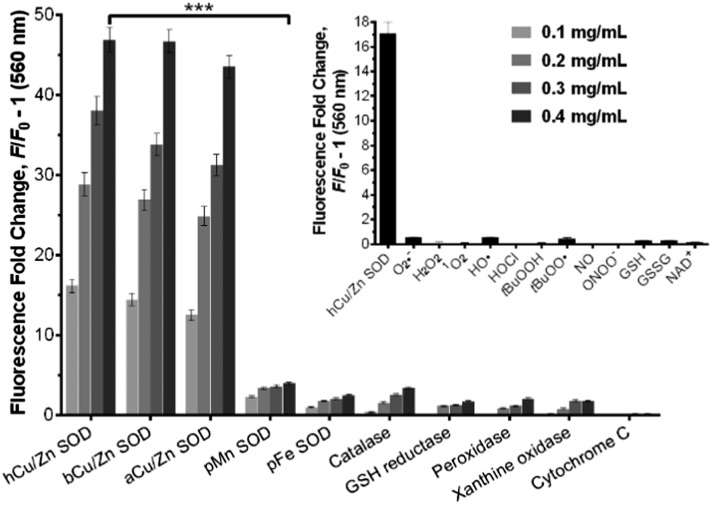
Fluorogenic response of **SODO** upon binding to different proteins (at 0.1, 0.2, 0.3 and 0.4 mg mL^–1^) and metabolites (inset, ROS & RNS: 100 μM) in 20 mM Tris-HCl buffer (pH = 7.4). *λ*_exc._: 460 nm, *λ*_em._: 560 nm. Values are represented as means and error bars as standard deviations (*n* = 3), *** for *p* < 0.001.

In view of the high selectivity and fluorogenic properties of **SODO**, we employed it to visualise changes in the expression of Cu/Zn SOD *in vivo*. We used **SODO** to image Cu/Zn SOD during the onset of inflammatory processes in zebrafish embryos.^9^ We employed a zebrafish tail fin injury model of inflammation by amputating the tail fin of embryos at 3 days post fertilization (dpf),^24^ which allowed us to examine the *in vivo* fluorogenic response of **SODO** in the inflammatory milieu. As shown in Fig. 3b, zebrafish undergoing inflammation displayed bright fluorescence in the wound margins (white arrows), which correspond to inflamed areas where Cu/Zn SOD is highly expressed. High magnification images corroborated the expression of Cu/Zn SOD in the cytoplasm of epithelial cells (Fig. 3c). We further confirmed these results by measuring the levels of the *sod1* gene before and after wounding using semi-quantitative reverse transcription-polymerase chain reaction (RT-PCR). As shown in Fig. 3d, the *sod1* gene was highly upregulated 5 h after wounding, in agreement with the fluorescence emission profile of **SODO** *in vivo* (Fig. S8 in ESI†). We also observed that **SODO** brightly stained oxidatively-stressed fibroblasts (Fig. S9 in ESI†), containing high levels of Cu/Zn SOD.^25^ Cell viability assays in fibroblasts also corroborated the marginal cytotoxicity of **SODO** within the working concentration range (Fig. S10 in ESI†).

**Fig. 3:**
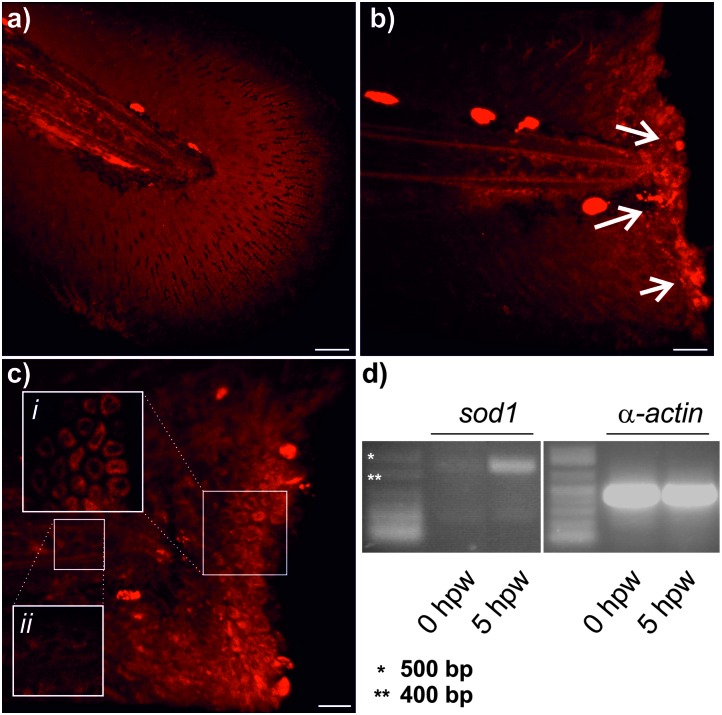
*In vivo* imaging of Cu/Zn SOD in inflamed zebrafish after treatment with **SODO** (10 μM). (a) Unwounded tail fin of a zebrafish embryo (3 dpf). (b) tail fin of a zebrafish embryo (3 dpf) 5 h after wounding (5 hpw). Strong fluorescence emission is observed towards the wound margin (white arrows). Bright spots in (a) and (b) away from the wound edge correspond to auto-fluorescence signals from pigment cells. (c) High magnification images showing bright fluorescence from **SODO** at the wound margin (i) compared to non-fluorescent unwounded areas (ii). (d) Semi-quantitative RT-PCR of *sod1* and *α-actin* genes at 0 and 5 hpw with corresponding ladders. Scale bars (a and b): 40 μm; (c): 20 μm.

In order to determine the binding mode of **SODO** in Cu/Zn SOD, we performed docking calculations to analyse the interaction between **SODO** at hCu/Zn SOD. Cu/Zn-SOD is found in all eukaryotic species as a homodimeric enzyme of ∼32 kDa containing one Cu and one Zn ion in each of the subunits, which are stabilized by an intra-chain disulfide bond.^26^ Our model predicted the interaction of **SODO** at the interface of the two subunits of Cu/Zn SOD (Fig. 4a). The binding at this conserved hydrophobic pocket, which is away from the catalytic site of the enzyme, is consistent with the previously observed species-independent response of **SODO** (Fig. 2) and the fact that the enzymatic activity of Cu/Zn SOD remained unaffected by **SODO** (Fig. S7 in ESI†). A closer examination of the binding revealed four hydrogen bonds between **SODO** and hCu/Zn SOD: one hydrogen bond between the oxygen atom of the ethoxy group and Val148, two hydrogen bonds between the nitrogen atoms of the triazole ring and the residues Lys9 and Asn53, and a final hydrogen bond between the *meso*-aniline group and Asp11 (Fig. 4b). The binding analysis suggests that the fluorogenic response of **SODO** is the result of combining the restriction in the rotation of the fluorophore by forming four hydrogen bonds and the deactivation of the quenching PeT due to the migration to a hydrophobic environment, as observed in our results from the *in vitro* characterisation assays.

**Fig. 4:**
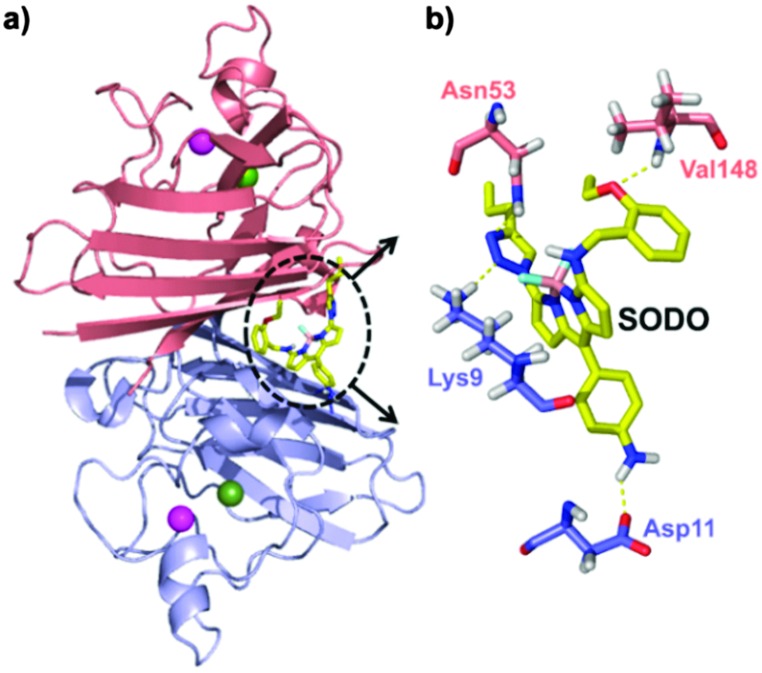
Molecular docking for the binding of **SODO** at hCu/Zn-SOD. (a) Illustration of the binding site of **SODO** (yellow) at the interface between the two monomeric subunits (blue and pink) of hCu/Zn-SOD (Cu and Zn are shown as green and magenta spheres, respectively). (b) Suggested hydrogen bonding interactions between **SODO** and different residues of hCu/Zn SOD.

In order to corroborate this hypothesis, we prepared two derivatives of **SODO** lacking the chemical groups involved in the interaction with hCu/Zn SOD (Fig. 5). We synthesized **SODO1** as the derivative without the ethoxy group in the amine ‘arm’ and **SODO2** as the derivative lacking the triazole nitrogen atoms (ESI† for synthetic details and characterisation), and compared their fluorogenic response to **SODO** after binding to hCu/Zn SOD. **SODO1** and **SODO2** showed remarkably lower fluorescence emission than **SODO**, confirming the relevance of both ethoxy and triazole groups for binding at hCu/Zn SOD (Fig. 5). These results confirmed the need of four hydrogen bonds, which are missing in the analogues **SODO1** and **SODO2** (Fig. S11 in ESI†), to restrict the torsional motion of **SODO** and induce its maximal fluorogenic response.

**Fig. 5:**
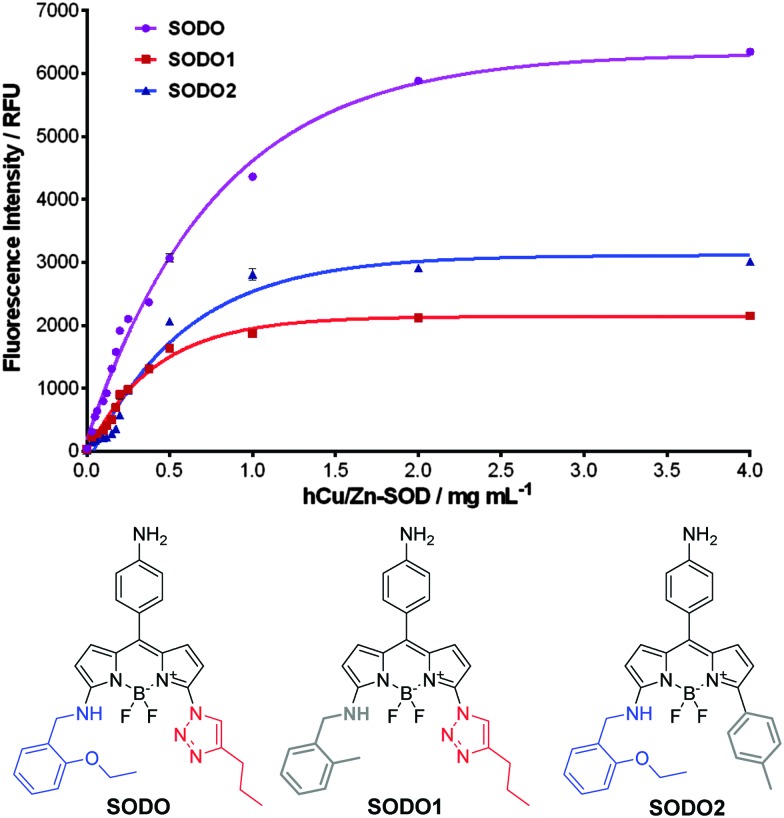
Fluorogenic response of **SODO** derivatives upon incubation with serial concentrations of hCu/Zn SOD in 20 mM Tris-HCl buffer (pH = 7.4). *λ*_exc._: 460 nm for **SODO** and **SODO1**, 510 nm for **SODO2**. *Φ*_F_ in hCu/Zn SOD: **SODO**: 0.45, **SODO1**: 0.11, **SODO2**: 0.22. Values are represented as means and error bars as standard deviations (*n* = 3).

In summary, we have designed a new class of BODIPY fluorogens with enhanced spectral properties by incorporating both rotational restriction and PeT-quenching groups. These new BODIPY fluorogens show excellent properties as polarity probes with minimal background emission in aqueous media and long Stokes shifts upon fluorescence activation. *In vitro* studies identified one derivative (**SODO**) as a highly selective fluorogenic probe for Cu/Zn SOD. **SODO** shows remarkable fluorescence emission only after binding to Cu/Zn SOD with very high selectivity over ROS-related enzymes and metabolites as well as the other SOD isoforms (*i.e.* Mn-SOD and Fe-SOD). The high selectivity of **SODO** enabled its use for imaging Cu/Zn SOD *in vivo* during the onset of an inflammatory response in a zebrafish tail fin injury model. Furthermore, we performed computational modelling to analyse the binding of **SODO** at Cu/Zn SOD. Structure–activity studies suggest that the binding occurs at the interface of the two enzymatic subunits and involves four residues to restrict the torsional motion of the BODIPY fluorophore and deactivate its PeT-quenching groups. **SODO** is the first fluorogenic probe for Cu/Zn SOD and represents a unique probe for the detection and *in vivo* imaging of Cu/Zn SOD during the progression of inflammatory disorders.

L. Z. acknowledges the ‘973’ program (2014CB932002), the Natural Science Foundation of China (11105150) and the Special Financial Grant from China Postdoctoral Science Foundation (2013T60613). J. C. E. acknowledges a NGS scholarship. Y. F. is a Wellcome Trust Sir Henry Dale Fellow (WT 100104/Z/12/Z). Y.-T. C. acknowledges funding from the National Medical Research Council (NMRC/CBRG/0015/2012). M. V. acknowledges funding from Medical Research Council, Marie Curie Career Integration Grant (333487) and the WT Institutional Strategic Support Fund.
