# Supplementary material for: A highly selective fluorogenic probe for the detection and in vivo imaging of Cu/Zn superoxide dismutase
Source: Chem Commun (Camb). 2016 Mar 4;52(58):9093–6. doi: 10.1039/c6cc00095a (PMC4943070; doi:10.1039/c6cc00095a)
Supplement: Supplementary file 1 [file CC-052-C6CC00095A-s001.pdf]

Electronic Supplementary Material (ESI) for ChemComm.
This journal is © The Royal Society of Chemistry 2016

Electronic Supporting Information

A Highly Selective Fluorogenic Probe for the Detection and *in vivo* Imaging of Cu/Zn Superoxide Dismutase

Liyun Zhang, Jun Cheng Er, Hao Jiang, Xin Li, Zhaofeng Luo, Thomas Ramezani, Yi Feng, Mui Kee Tang, Young-Tae Chang and Marc Vendrell

Table of Contents

A. Supporting Tables and Figures S2

B. Experimental Procedures S17

C. NMR Spectra S24

D. References S34

A. Supporting Tables and Figures

Table S1. Chemical structures and characterisation for the **MK** fluorogens in DMSO.

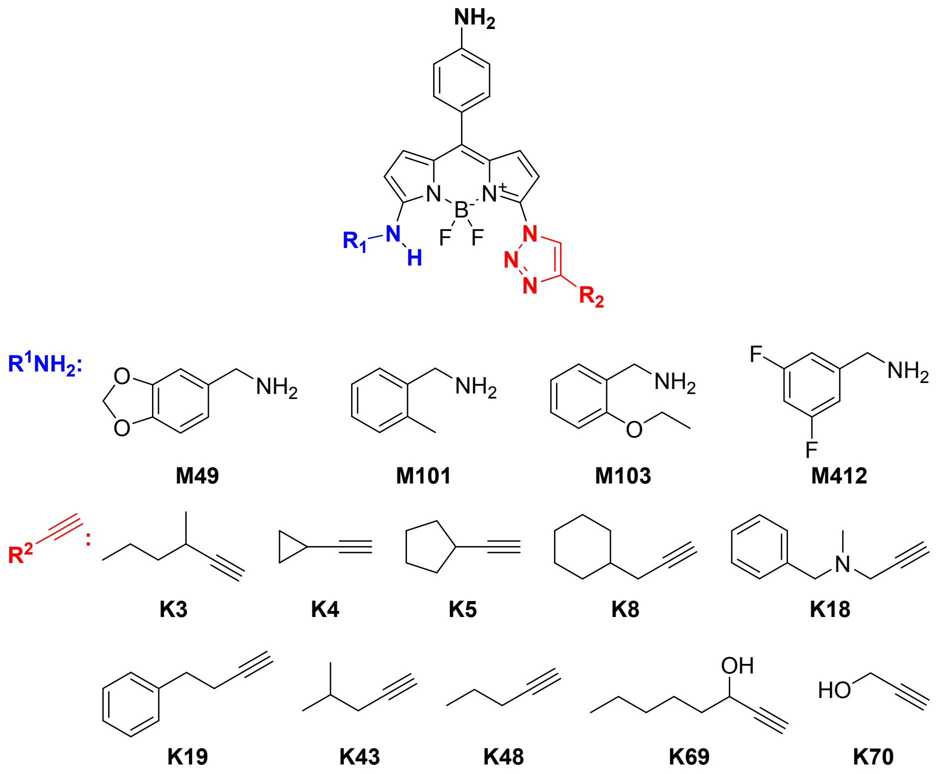

Code	Structure	HPLC purity	*m/z* calc., $[M+H]^+$	*m/z* found, $[M+H]^+$	λ_{max} Abs. (nm)	λ_{max} Em. (nm)	Φ_F
**MK49-3**	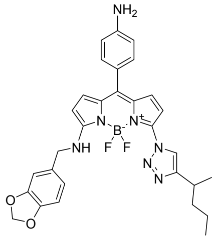	98%	570.3	570.7	474	565	0.014
**MK49-4**	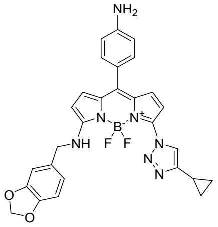	98%	540.2	540.6	474	566	0.014
**MK49-5**	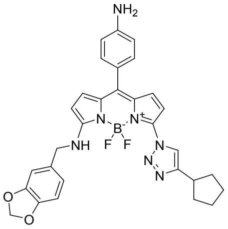	98%	568.3	568.7	475	563	0.014
**MK49-8**	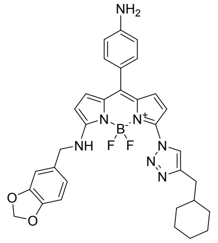	98%	596.3	596.7	474	565	0.014
**MK49-18**	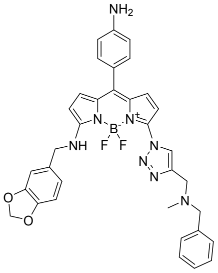	98%	633.3	633.7	473	563	0.011

Code	Structure	HPLC purity	*m/z* calc., $[M+H]^+$	*m/z* found, $[M+H]^+$	λ_{max} Abs. (nm)	λ_{max} Em. (nm)	Φ_F
**MK49-19**	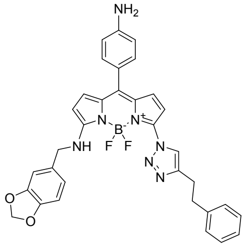	98%	604.3	604.7	474	564	0.015
**MK49-43**	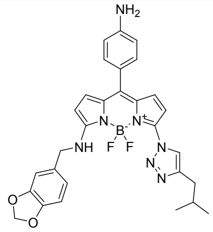	98%	556.3	556.7	475	566	0.014
**MK49-48**	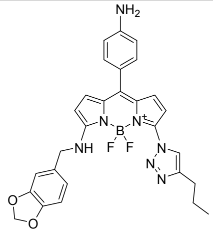	98%	542.2	542.6	475	563	0.014
**MK49-69**	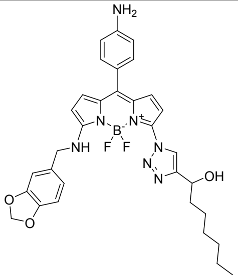	98%	600.3	600.7	473	563	0.012
**MK49-70**	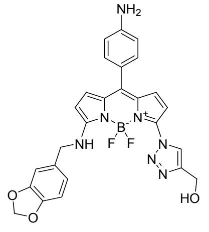	98%	530.2	530.6	474	564	0.012

Code	Structure	HPLC purity	*m/z* calc., $[M+H]^+$	*m/z* found, $[M+H]^+$	λ_{max} Abs. (nm)	λ_{max} Em. (nm)	Φ_F
**MK101-3**	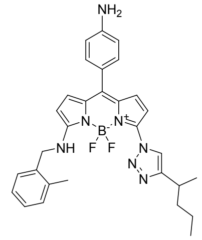	97%	540.3	540.7	475	563	0.015
**MK101-4**	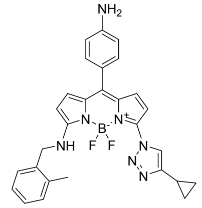	98%	510.2	510.6	474	565	0.016
**MK101-5**	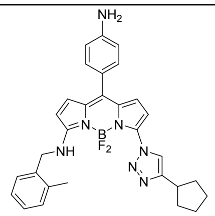	97%	538.3	538.6	475	565	0.016
**MK101-8**	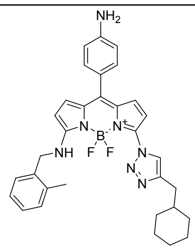	94%	566.3	566.7	475	564	0.016
**MK101-18**	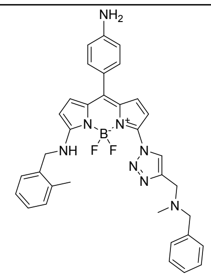	92%	603.3	603.7	474	563	0.012
**MK101-19**	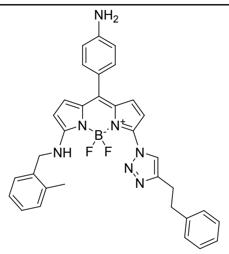	82%	574.3	574.7	475	564	0.016

Code	Structure	HPLC purity	*m/z* calc., $[M+H]^+$	*m/z* found, $[M+H]^+$	λ_{max} Abs. (nm)	λ_{max} Em. (nm)	Φ_F
**MK101-43**	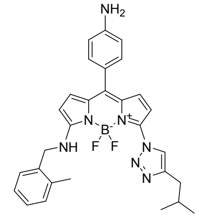	90%	526.3	526.7	474	563	0.015
**MK101-48**	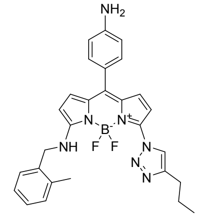	90%	512.3	512.6	473	563	0.015
**MK101-69**	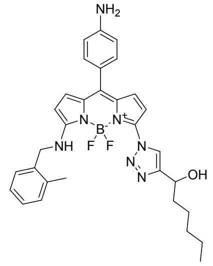	96%	570.3	570.7	475	563	0.014
**MK101-70**	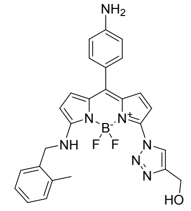	87%	500.2	500.6	474	565	0.014
**MK103-3**	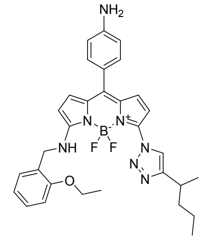	99%	570.3	570.7	474	564	0.014
**MK103-4**	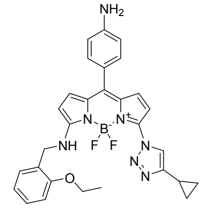	95%	540.2	540.6	473	563	0.015

Code	Structure	HPLC purity	*m/z* calc., $[M+H]^+$	*m/z* found, $[M+H]^+$	λ_{max} Abs. (nm)	λ_{max} Em. (nm)	Φ_F
**MK103-5**	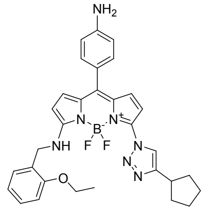	99%	568.3	568.6	473	564	0.014
**MK103-8**	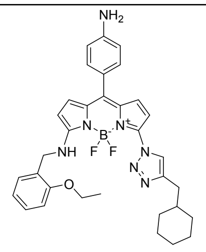	98%	596.3	596.7	473	563	0.015
**MK103-18**	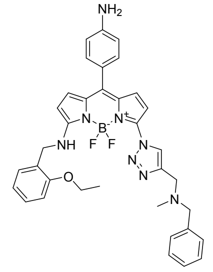	95%	633.3	633.7	473	565	0.012
**MK103-19**	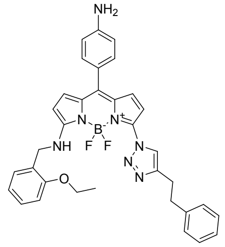	95%	604.3	604.7	475	565	0.016
**MK103-43**	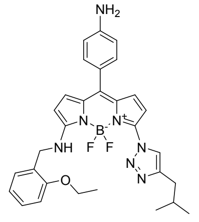	97%	556.3	556.7	474	563	0.015
**MK103-48**	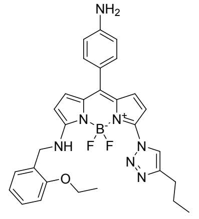	96%	542.3	542.7	475	563	0.014

Code	Structure	HPLC purity	*m/z* calc., $[M+H]^+$	*m/z* found, $[M+H]^+$	λ_{max} Abs. (nm)	λ_{max} Em. (nm)	Φ_F
**MK103-69**	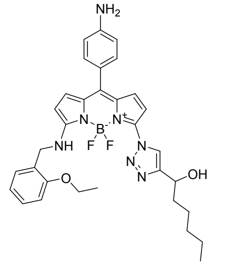	93%	600.3	600.7	474	563	0.014
**MK103-70**	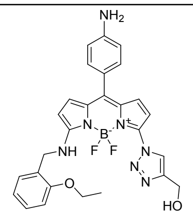	98%	530.2	530.6	474	563	0.014
**MK412-3**	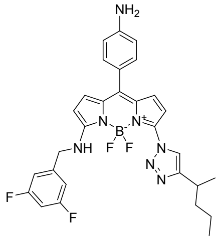	98%	562.3	562.7	475	563	0.015
**MK412-4**	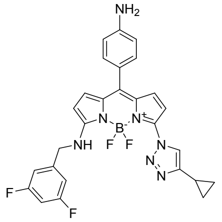	98%	532.2	532.6	475	563	0.016
**MK412-5**	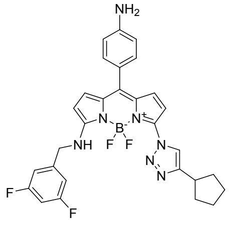	98%	560.2	560.6	476	565	0.016
**MK412-8**	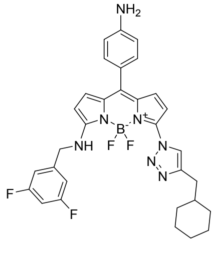	98%	588.3	588.7	476	563	0.015

Code	Structure	HPLC purity	*m/z* calc., $[M+H]^+$	*m/z* found, $[M+H]^+$	λ_{max} Abs. (nm)	λ_{max} Em. (nm)	Φ_F
**MK412-18**	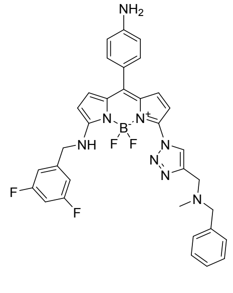	98%	625.3	625.7	473	563	0.01
**MK412-19**	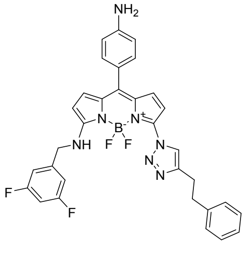	98%	596.2	596.6	475	564	0.014
**MK412-43**	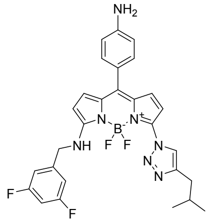	98%	548.2	548.6	476	564	0.016
**MK412-48**	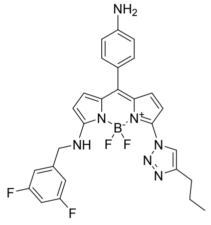	97%	534.2	534.6	474	563	0.016
**MK412-69**	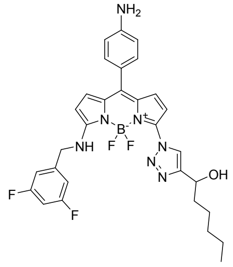	98%	592.3	592.6	475	563	0.014

Code	Structure	HPLC purity	*m/z* calc., $[M+H]^+$	*m/z* found, $[M+H]^+$	λ_{max} Abs. (nm)	λ_{max} Em. (nm)	Φ_F
**MK412-70**	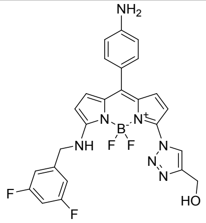	88%	522.2	522.5	474	563	0.013

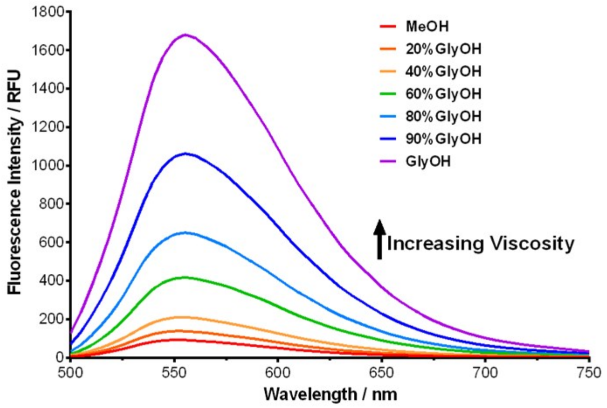

Figure S1. Effect of solvent viscosity on the fluorescence emission of **MK103-48 (SODO**). Emission spectra of **MK103-48** (10 µM) at r.t. in mixtures of methanol (MeOH) and glycerol (GlyOH)) with increasing viscosity.

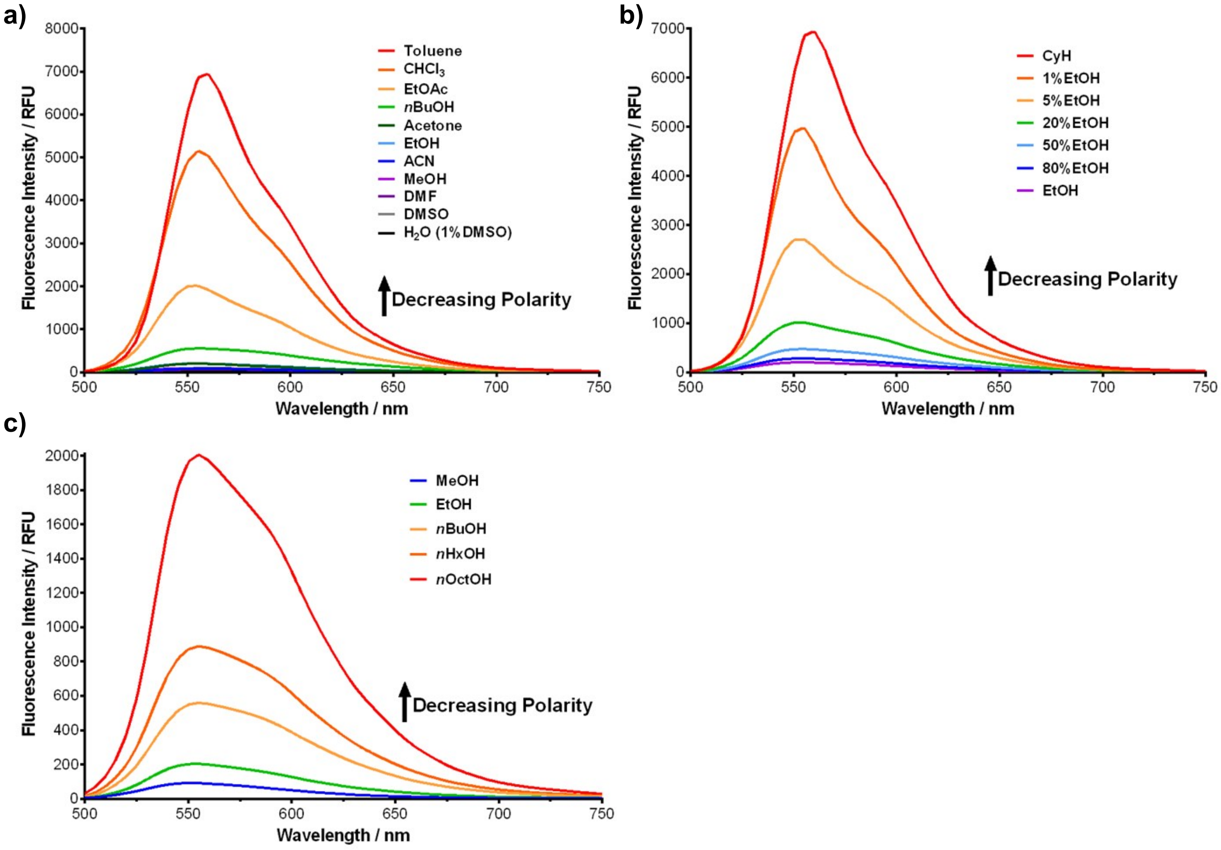

Figure S2. Effect of solvent polarity on the fluorescence emission of **MK103-48 (SODO)**. Emission spectra of **MK103-48** (10 µM) in a) various solvents, b) various ratios of cyclohexane (CyH) and ethanol (EtOH), c) various primary alcohols of increasing chain length.

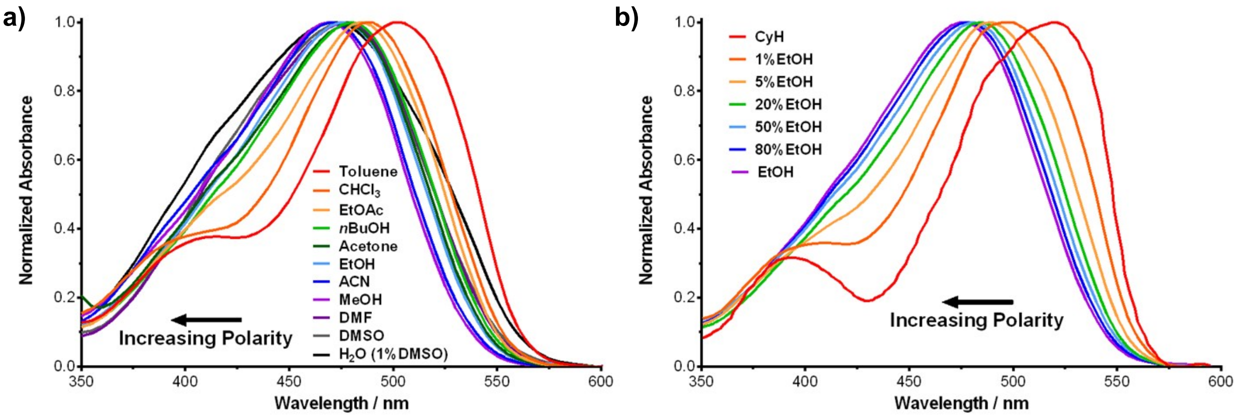

Figure S3. Effect of solvent polarity on the absorbance spectra of **MK103-48 (SODO)**. Absorbance spectra of **MK103-48** (10 μM) in a) various solvents, b) various ratios of cyclohexane (CyH) and ethanol (EtOH).

Table S2. Photophysical properties of **MK** fluorogens in environments of increasing polarity.*

Solvent	Dielectric constant (ε)	$\lambda_{abs.\ max}$ (nm)	$\lambda_{em.\ max}$ (nm)	Φ_F
Cyclohexane	2.02	524	560	0.74
Toluene	2.38	505	560	0.73
Chloroform	4.81	490	560	0.55
n-Octanol	3.40	486	560	0.27
Ethyl acetate	6.02	485	560	0.22
n-Hexanol	13.3	483	560	0.13
n-Butanol	18.0	480	560	0.080
Acetone	21.0	480	560	0.024
Ethanol	24.6	480	560	0.027
Methanol	33.0	471	560	0.012
Acetonitrile	37.5	470	560	0.011
Dimethylformamide	38.0	480	560	0.010
Dimethylsulfoxide	46.7	480	560	0.014
Water (1% DMSO)	80.1	475	620	0.007

* Values determined for the representative derivative **MK103-48** (**SODO**) at 10 μM in r.t.

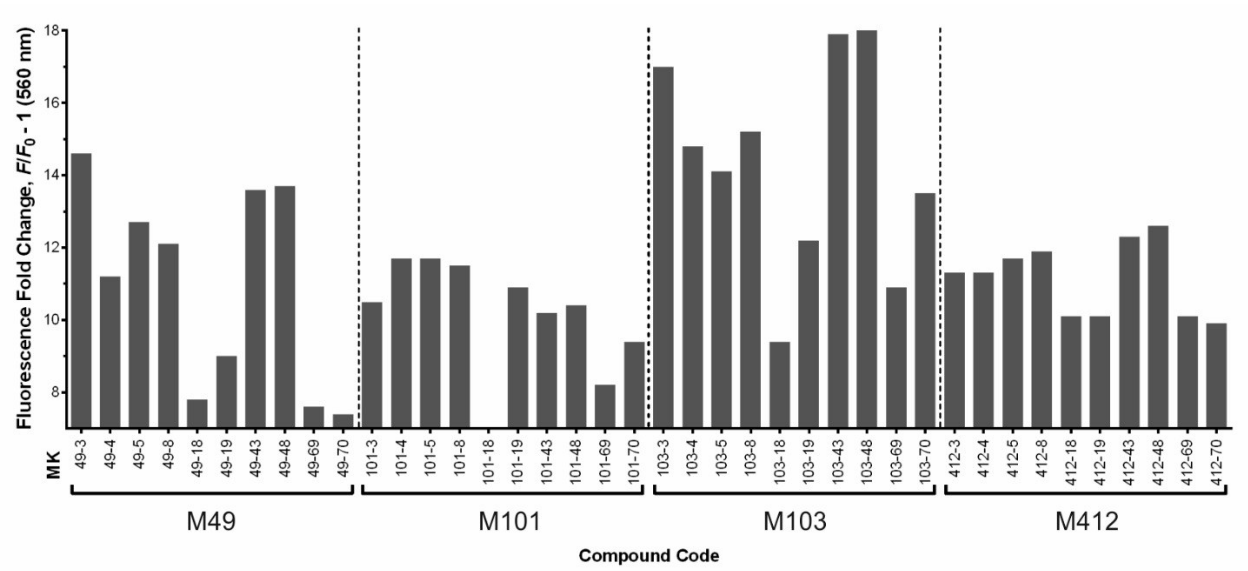

Figure S4. Fluorescence response of **MK** fluorogens upon incubation with hCu/Zn SOD in 20 mM Tris-HCl buffer (pH = 7.4). $\lambda_{exc.}$: 460 nm, $\lambda_{em.}$: 560 nm.

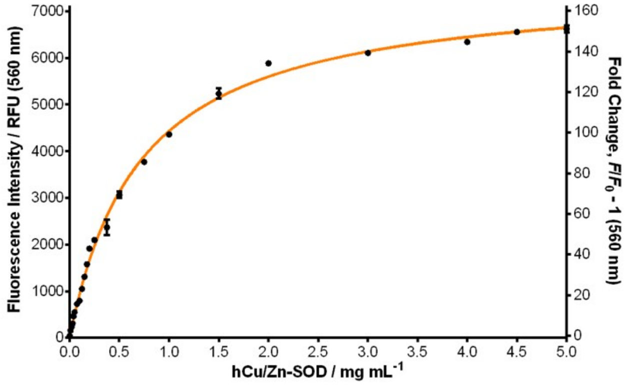

Figure S5. Fluorescence response of **SODO** (10 μM) at 560 nm upon incubation with serial concentrations of hCu/Zn SOD in 20 mM Tris-HCl buffer (pH = 7.4). $\lambda_{exc.}$: 460 nm, $\lambda_{em.}$: 560 nm. Values are represented as means and error bars as standard deviations (n = 3). The limit of detection was determined as the mean value plus 3 times the standard deviation of the blank measurements (LOD = 10 μg mL^{-1}).

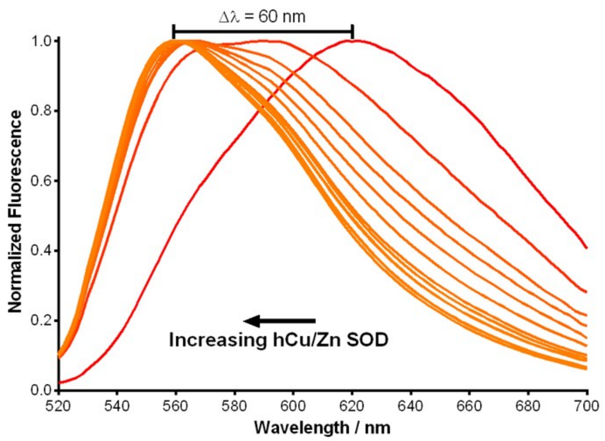

Figure S6. Normalized fluorescence spectra of **SODO** (10 μM) upon incubation with serial concentrations of hCu/Zn SOD (from 0.01 to 0.5 mg mL^{-1}) in 20 mM Tris-HCl buffer (pH = 7.4). $\lambda_{exc.}$: 460 nm.

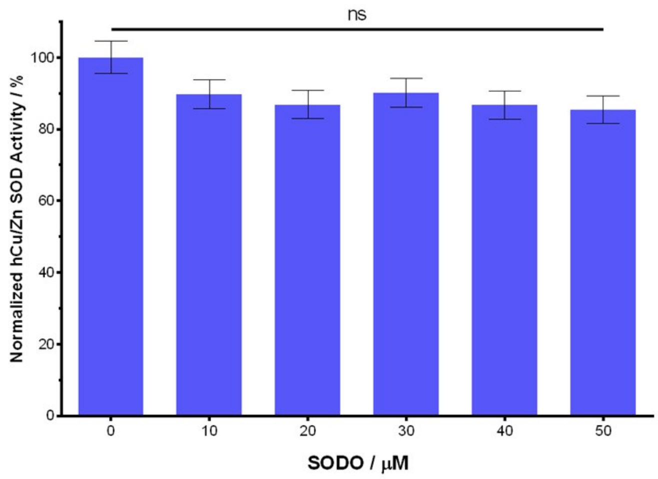

Figure S7. Activity assay of hCu/Zn SOD in the presence of **SODO** (0 – 50 μM). The activity of hCu/Zn SOD was determined at 37 °C using the xanthine/xanthine oxidase method based on the production of $O_2{\bullet}^-$ anions.[1] Values are represented as means ± s.d. (n=3). No significant differences ($p > 0.05$) were determined between the control and any of the treatments.

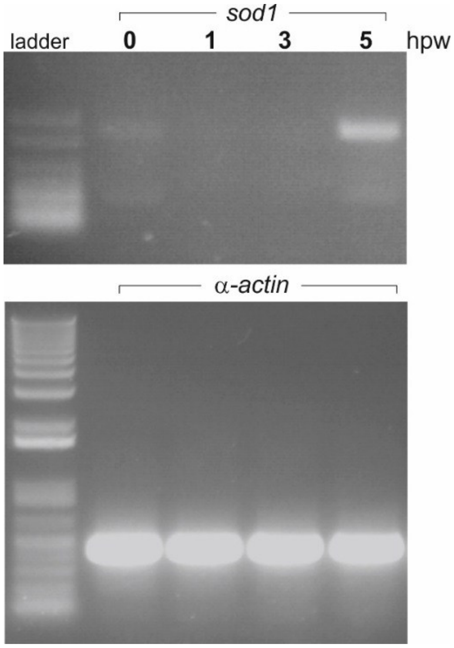

Figure S8. Semi-quantitative RT-PCR analysis for *sod1* and *α-actin* genes in wounded regions from zebrafish at different time points (0, 1, 3 and 5 hpw) with their corresponding ladders.

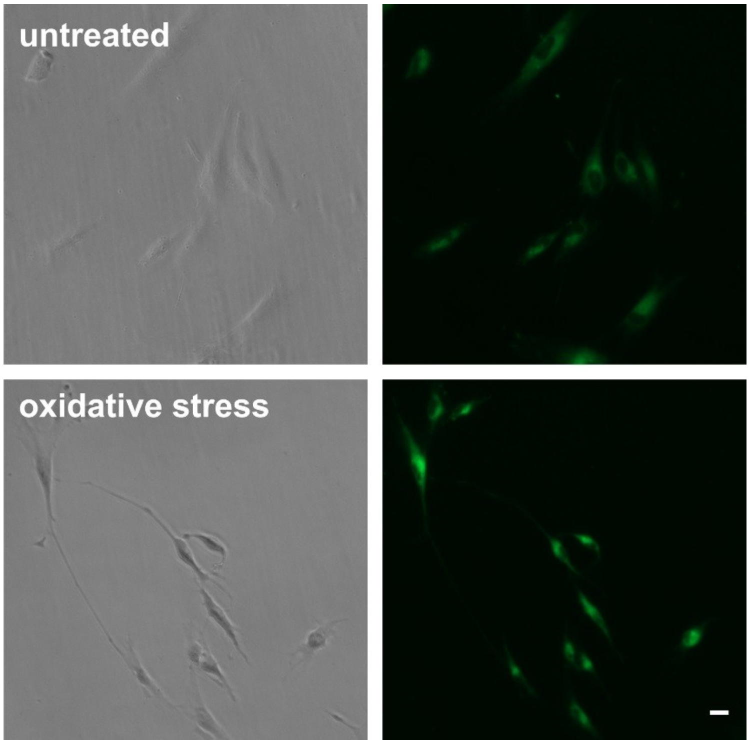

Figure S9. Images of normal and oxidatively-stressed primary human fibroblasts[1] after treatment with **SODO**. **SODO** (1 μM) was incubated for 30 min, and cells were visualised under an epifluorescence microscope to acquire the corresponding brightfield (*left*) and fluorescence (*right*) images. Scale bar: 20 μm.

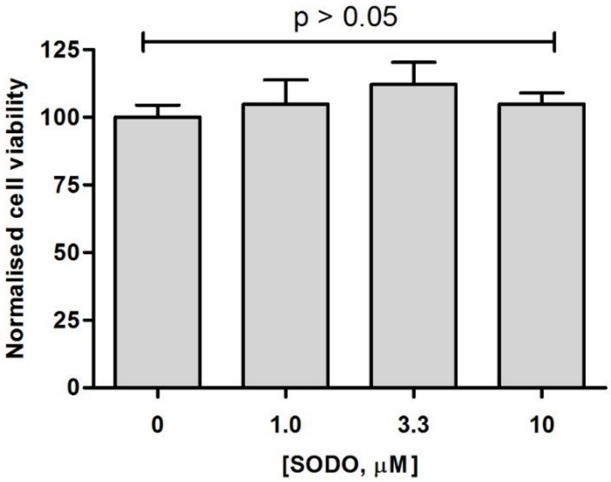

Figure S10. Cell viability of primary human fibroblasts after incubation with different concentrations of **SODO**. Data represented as means ± s.e.m. (n=4). No significant differences ($p > 0.05$) were determined between the control and any of the treatments.

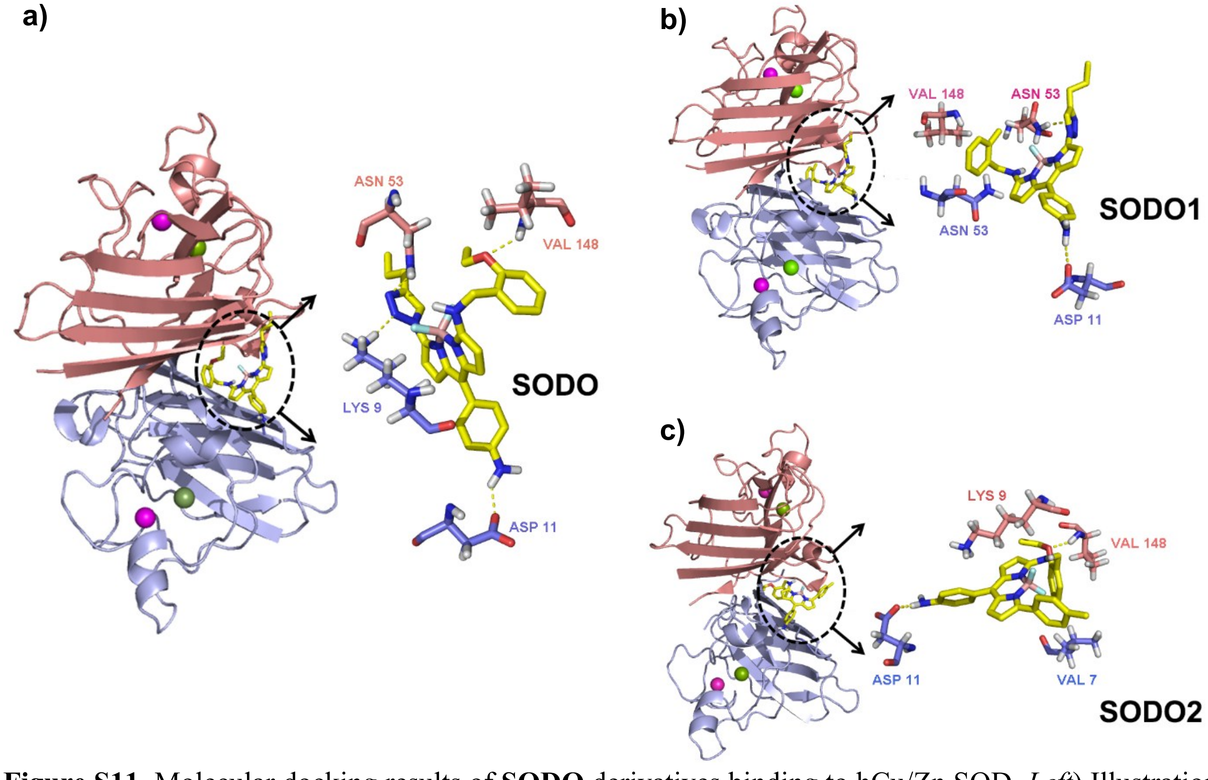

Figure S11. Molecular docking results of **SODO** derivatives binding to hCu/Zn SOD. *Left*) Illustration of the binding site of **SODO** derivatives (yellow) at the interface between the two monomeric subunits (blue and pink) of hCu/Zn-SOD (Cu and Zn are shown as green and magenta spheres, respectively). *Right*) Suggested hydrogen bonding pattern between **SODO** analogues and the different residues of hCu/Zn SOD. Data presented for a) **SODO**, b) **SODO1** and c) **SODO2**.

B. Experimental Procedures

Materials

All commercially available reagents, solvents and proteins were purchased from Sigma Aldrich, Alfa Aesar or TCI and used as received unless otherwise stated. Anhydrous solvents were purchased from Alfa Aesar and used without further purification.

Spectral measurements

UV/Vis absorption spectra of protein were recorded on a Hitachi U-2801 spectrophotometer from 200 to 1000 nm. All other spectroscopic data (absorption and fluorescence) were measured on a SpectraMax M2 spectrophotometer (Molecular Devices). Spectroscopic data analysis was performed using GraphPad Prism 6.0.

^{1}H and ^{13}C spectra were acquired on Bruker DRX500 and AV500 spectrometers respectively (500 MHz ^{1}H; 126 MHz ^{13}C) using the Bruker TopSpin™ software. Data were processed using MestreNova and reported in δ (ppm). ^{1}H NMR spectra were referenced to solvent residual signals as the ^{1}H internal reference; ^{13}C NMR spectra were referenced to solvent resonances as the ^{13}C internal reference. Analytical characterisation was performed on a LC-MS (Agilent-1200 series) with a DAD detector and a single quadrupole mass spectrometer (6130 series) with an ESI probe; analytical HPLC method: eluents, A: H_2O (0.1% HCOOH), B: CH_3CN (0.1% HCOOH), gradient 5% B to 95% B (10 min); reverse-phase Phenomenex C_{18} Luna column (4.6 x 50 mm^2, 3.5 µm particle size), flow rate: 1 mL/min. High resolution mass spectra (ESI) were obtained on a Finnigan/MAT 95XL-T spectrometer.

Quantum yield calculations

Quantum yields were calculated by measuring the integrated emission area of the fluorescence spectra in its respective solutions and comparing to the area measured for Acridine Yellow ($\Phi_F = 0.47$) in EtOH (n = 1.361). Quantum yields were calculated using the equation:

$$\Phi_F^{sample} = \Phi_F^{reference}\left(\frac{F^{sample}}{F^{reference}}\right)\left(\frac{n^{sample}}{n^{reference}}\right)^2\left(\frac{f^{reference}}{f^{sample}}\right)$$

where F is the integrated intensities of the emission spectra, n is the refractive index of the solvent, f is absorption factor ($f = (1 - 10^{-A})$) and A is the absorbance at the excitation wavelength selected for reference and samples. Emission was integrated between 480 and 750 nm.

Expression and purification of hCu/Zn SOD

The expression plasmids were generous gifts from Professor Wang Guanghui.[2] Proteins were expressed in *Escherichia coli* strain *BL21* (DE3) and affinity purified using His·Bind Resin (Novagen) according to the manufacturer's protocol. Further purification was performed by gel filtration using sephacryl S-200 (HiPrep 16/60 Sephacryl S-200 HR). Protein purity was confirmed using SDS-PAGE analysis and the concentration of Cu/Zn SOD was calculated from the absorption coefficient at 280 nm (ε = 0.364) and the relative molecular weight (Mw ≈ 32 kDa). The final protein was buffered in Tris-HCl (20 mM, pH 7.4) containing 0.1 M NaCl. The Cu/Zn SOD activity was verified with a superoxide assay kit (Nanjing Jiancheng Bioengineering Institute, Nanjing, China) using the xanthine/xanthine oxidase method based on the production of $O_2{\bullet}^-$ anions.[3]

Molecular modelling

The crystallographic coordinates of the 1.80 Å hCu/Zn SOD structures were obtained from the protein data bank (PDB ID 1HL5), processed using Reduce, and manually checked.[4, 5] The **SODO** structure was optimised using the Gaussian03 program (B3LYP/6-31G* level). AutoDock Vina[6] and AutoDock Tools were used for the molecular docking. Each of the 9 biological assemblies in the 1HL5 structures were used for docking. For each of them, a search space was chosen to include all the atoms of the protein dimer. The calculations with the exhaustiveness parameter of 256, 512 or 1024 were all performed. All 243 resulting conformations were analysed in AutoDock Tools, and the conformation with the lowest energy was chosen for further analysis. All figures were rendered using PyMOL v0.99. (http://www.pymol.org).

In vivo imaging

The tailfins of zebrafish embryos were amputated at 3 days post fertilization (dpf). The embryos were transferred into a petri dish containing 10 mL of staining solution (0.3 x Danieau solution, 10 μM **SODO**, 0.1% DMSO). Embryos were incubated in the dark at 28.5 ºC for 3 – 4 h. The staining solution was removed and the embryos were washed 2 – 3 times with 0.3x Danieau solution and embedded in 1% low melting point agarose for imaging. Embryos were imaged 5 – 6 h post wounding (hpw) using an inverted Zeiss LSM780 confocal microscope. Images were analysed and processed using Imaris.

Cell culture and cell viability assays

Primary human fibroblasts were kindly provided by Dr. Brian McHugh (University of Edinburgh) and grown using Dulbecco's Modified Eagle Medium (DMEM) supplemented with 10% fetal bovine serum (FBS), antibiotics (100 U mL^{-1} penicillin and 100 mg mL^{-1} streptomycin) and 2 mM L-glutamine in a humidified atmosphere at 37 °C with 5% CO_2. Oxidative stress was induced using H_2O_2 as previously reported.[1]

Cell viability was determined with a TACS® MTT Cell Proliferation assay (Trevigen) according to the manufacturer's instructions. Briefly, human fibroblasts cells were plated on 96-well plates the day before the experiment, reaching around 60-80% confluence on the day of the experiment. **SODO** was added at different concentrations and cells were incubated at 37 ºC for 2 h. Afterwards, cells were washed, treated according to the manufacturer's instructions and their absorbance (570 nm) was measured in a spectrophotometer. Cell viability data was normalised to the proliferation of cells without addition of **SODO**.

Semi-quantitative RT-PCR

RNA of unwounded (0 hpw) and wounded zebrafish embryos (3 dpf) were isolated according to the TRIZOL® Reagent (Life Technologies) manufacturer's protocol and cDNA was synthesized with SuperScript II Reverse Transcriptase (Life Technologies) following the manufacturer's protocol. Equal

amounts of RNA from the different time points were used in the cDNA synthesis reactions. For PCR amplification of *sod*1 and *α-actin*, One Taq® Polymerase (NEB) was used following the manufacturer's protocol with an annealing temperature of 60°C and 30 cycles.

*sod*1 forward:	5'-ATGGTGAACAAGGCCGTTTGTGTGC
*sod*1 reverse:	5'-TCACTGAGTGATGCCGATCACTCC
α-actin forward:	5'-ACCACCGGTATTGTGCTGGATGC
α-actin reverse:	5'-ACAGATCCTTACGGATGTCAATGTC

Chemical synthesis

General procedure for the preparation of MK fluorogens. The resin loaded 3,5-dichloro-4,4-difluoro-8-(4-aminophenyl)-4-bora-3*a*,4*a*-diaza-*s*-indacene[7] (63 mg, 21 μmol) was swollen in CH_2Cl_2 for 10 min and filtered. A suspension of NaN_3 (13 mg, 0.19 mmol, 9 eq.) in DMF (1.0 mL) was added and the reaction suspension shaken at r.t. for 30 min. After washing with DMF (4 × 5 mL), a solution of the respective amine (ca. 50 μL, 0.32 mmol, 15 eq.), DIEA (0.15 mL) in DMF (0.8 mL) was added and the reaction shaken at rt for 1 h. The alkyne (ca. 30 μL, 0.30 mmol, 10 eq.), CuI (17 mg, 86 μmol, 4 eq.) and ascorbic acid (15 mg, 86 μmol, 4 eq.) were subsequently added and the suspension further shaken for another 30 min. The resin was filtered, washed with DMF (4 × 5 mL) and CH_2Cl_2 (2 × 5 mL) following which cleavage was performed with 0.5% TFA in CH_2Cl_2 (3 × 5 mL, 10 minutes each). The crude solution was extracted with sat. $NaHCO_3$ (3 × 15 mL) and the organic extracts recovered, concentrated and purified by flash column chromatography on silica gel (eluent: CH_2Cl_2 to CH_2Cl_2:MeOH (98:2)) to afford the **MK** compounds as orange solids.

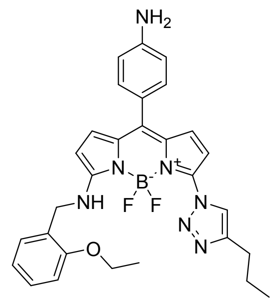

SODO (MK103-48)

3-((2-ethoxybenzyl)amino)-4,4-difluoro-5-(4-propyl-1*H*-1,2,3-triazol-1-yl)-8-(4-aminophenyl)-4-bora-3a,4a-diaza-*s*-indacene (SODO, MK103-48). (2.9 mg, 5.4 µmol, 26% yield); ^{1}H NMR (500 MHz, Acetone-d_6) δ 8.16 (s, 1H), 7.49 (d, *J* = 19.1 Hz, 1H), 7.37 (d, *J* = 7.4 Hz, 1H), 7.31 – 7.24 (m, 1H), 7.27 (d, *J* = 8.4 Hz, 2H), 7.14 (d, *J* = 5.1 Hz, 1H), 7.01 (d, *J* = 8.2 Hz, 1H), 6.92 (t, *J* = 7.4 Hz, 1H), 6.82 (t, *J* = 10.4 Hz, 2H), 6.70 (d, *J* = 5.1 Hz, 1H), 6.50 (t, *J* = 4.6 Hz, 2H), 5.12 (s, 2H), 4.76 (d, *J* = 6.5 Hz, 2H), 4.13 (q, *J* = 7.0 Hz, 2H), 2.73 (t, *J* = 7.6 Hz, 2H), 1.83 – 1.66 (m, 2H), 1.43 (t, *J* = 7.0 Hz, 3H), 1.02 (t, *J* = 7.4 Hz, 3H); ^{13}C NMR (126 MHz, Acetone-d_6) δ 163.2, 157.7, 151.1, 147.4, 137.2, 136.4, 134.4, 132.8, 132.6 132.4, 130.2, 129.58, 126.6, 124.0, 122.7, 121.3, 118.6, 114.7, 113.7, 112.3, 109.2, 64.5, 45.2, 28.3, 23.5, 15.1, 14.2; HRMS (ESI+) calcd. for $C_{29}H_{30}BF_2N_7O$, $[M+Na]^+$: 564.2470, found: 564.2481.

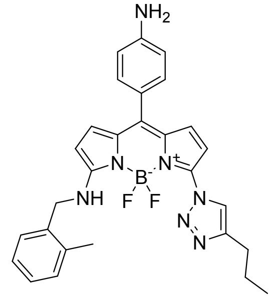

SODO1 (MK101-48)

3-((2-methylamino)-4,4-difluoro-5-(4-propyl-1*H*-1,2,3-triazol-1-yl)-8-(4-(4-aminophenyl)-4-bora-3a,4a-diaza-*s*-indacene (SODO1, MK101-48). (2.7 mg, 5.3 µmol, 25% yield); ^{1}H NMR (500 MHz, CD_2Cl_2) δ 8.02 (s, 1H), 7.32 (d, *J* = 8.4 Hz, 2H), 7.27 (d, *J* = 7.6 Hz, 1H), 7.26 – 7.18 (m, 3H), 7.08 (d, *J* = 5.0 Hz, 1H), 6.78 (d, *J* = 8.4 Hz, 2H), 6.55 (d, *J* = 4.0 Hz, 1H), 6.54 (d, *J* = 4.0 Hz, 1H), 6.52 (s, 1H), 6.29 (d, *J* = 5.0 Hz, 1H), 4.59 (d, *J* = 6.1 Hz, 2H), 4.02 (s, 2H), 2.74 (t, *J* = 7.6 Hz, 2H), 2.35 (s, 3H), 1.80 – 1.68

(m, 2H), 1.00 (t, J = 7.4 Hz, 3H); ^{13}C NMR (126 MHz, CD_2Cl_2) δ 162.5, 149.0, 147.7, 137.1, 136.7, 134.9, 134.02, 132.4, 132.3, 132.2, 131.3, 128.8, 128.0, 127.0, 123.9, 123.8, 119.7, 114.9, 114.8, 112.0, 109.6, 47.2, 28.2, 23.3, 19.4, 14.1; HRMS (ESI+) calcd. for $C_{28}H_{28}BF_2N_7$, $[M+Na]^+$: 534.2364, found: 534.2375.

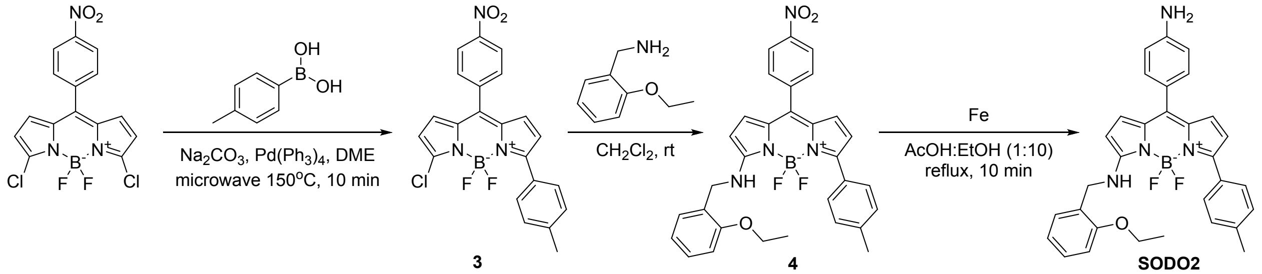

3-((2-ethoxybenzyl)amino)-4,4-difluoro-5-(*p*-tolyl)-8-(4-aminophenyl)-4-bora-3a,4a-diaza-*s*-indacene (SODO2). A solution of **4** (14 mg, 25 μmol) in a solvent mixture of EtOH and AcOH (10:1, 80 mL) was heated to reflux. A suspension of iron powder (28 mg, 0.50 mmol, 20 eq.) was activated in 1 M HCl for 1 min, rinsed with absolute EtOH and added to the hot reaction solution. The reaction mixture was monitored by TLC. Upon completion, the iron was removed and the solvent was evaporated *in vacuo*. The crude residue was redissolved in CH_2Cl_2 (30 mL) and extracted with saturated $NaHCO_3$ (3 × 30 mL). The residue was purified by flash column chromatography on silica gel to afford **SODO2** as a pale red solid. (12 mg, 22 μmol, 85% yield); ^{1}H NMR (500 MHz, CD_3CN) δ 7.67 (d, J = 8.2 Hz, 2H), 7.30 – 7.23 (m, 4H), 7.21 (d, J = 8.0 Hz, 2H), 7.04 (s, 1H), 7.01 (d, J = 4.9 Hz, 1H), 6.97 (d, J = 8.1 Hz, 1H), 6.91 (td, J = 7.5, 0.9 Hz, 1H), 6.73 (d, J = 8.5 Hz, 2H), 6.48 (d, J = 3.8 Hz, 1H), 6.42 (d, J = 4.9 Hz, 1H), 6.38 (d, J = 3.8 Hz, 1H), 4.57 (d, J = 6.7 Hz, 2H), 4.46 (s, 2H), 4.10 (q, J = 7.0 Hz, 2H), 2.37 (s, 3H), 1.41 (t, J = 7.0 Hz, 3H); ^{13}C NMR (126 MHz, CD_3CN) δ 162.5, 157.8, 150.4, 147.2, 138.4, 136.2, 135.3, 133.7, 133.5, 132.8, 132.8, 130.3, 129.8, 129.6, 129.6, 126.9, 124.0, 121.4, 115.6, 114.8, 112.7, 112.0, 64.8, 45.0, 21.3, 15.0; HRMS (ESI+) calcd. for $C_{31}H_{29}BF_2N_4O$, $[M+H]^+$: 523.2481, found: 523.2488.

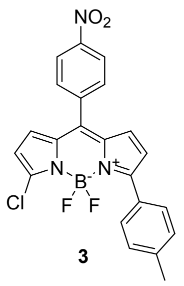

3

3-chloro-4,4-difluoro-5-(*p*-tolyl)-8-(4'-nitrophenyl)-4-bora-3a,4a-diaza-*s*-indacene (3). A solution of 3,5-dichloro-8-(4'-nitrophenyl)-4,4-difluoro-4-bora-3*a*,4*a*-diaza-*s*-indacene[6] (50 mg, 0.13 mmol, 1 eq.), *p*-tolylboronic acid (18 mg, 0.13 mmol, 1 eq.), Na_2CO_3 (42 mg, 0.39 mmol, 3 eq.) and $Pd(PPh_3)_4$ (15 mg, 0.013 mmol, 10 mol%) catalyst in DME (1 mL) was purged with argon gas for 10 min. The reaction mixture was stirred under microwave irradiation for 10 min at 150 ^oC and 300 W. The reaction was quenched by addition of water and the aqueous layer was extracted with CH_2Cl_2. The organic layers were dried with anhydrous Na_2SO_4, evaporated *in vacuo*, and purified by flash column chromatography on silica gel (eluent: CH_2Cl_2:Hexane (1:2) to afford **3** as a pale red solid (13 mg, 30 μmol, 23% yield); ^1H NMR (500 MHz, CD_2Cl_2) δ 8.38 (d, J = 8.6 Hz, 2H), 7.87 (d, J = 8.2 Hz, 2H), 7.75 (d, J = 8.6 Hz, 2H), 7.34 (d, J = 8.0 Hz, 2H), 6.89 (d, J = 4.5 Hz, 1H), 6.79 (d, J = 4.4 Hz, 1H), 6.72 (d, J = 4.1 Hz, 1H), 6.45 (d, J = 4.2 Hz, 1H), 2.45 (s, 3H); ^{13}C NMR (126 MHz, CD_2Cl_2) δ 163.1, 149.7, 143.0, 142.0, 140.7, 140.6, 140.2, 137.5, 133.3, 133.0, 132.1, 130.2, 129.8, 129.5, 129.4, 124.2, 123.3, 118.8, 21.9; HRMS (APCI+) calcd. for $C_{22}H_{16}BClF_2N_3O_2$, $[M+H]^+$: 438.0991, found: 438.0986.

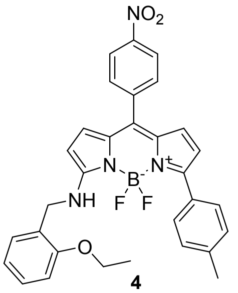

4

3-((2-ethoxybenzyl)amino)-4,4-difluoro-5-(*p*-tolyl)-8-(4-nitrophenyl)-4-bora-3a,4a-diaza-*s*-indacene (4). A solution of **3** (13 mg, 30 μmol, 1 eq.) and 2-ethoxybenzylamine (6.5 μL, 60 μmol, 2 eq.) in CH_2Cl_2

was stirred for 4 h until reaction completion. The mixture was purified by flash column chromatography on silica gel (eluent: CH_2Cl_2:Hex (1:2) to afford **4** as a pale red solid (14 mg, 26 μmol, 86% yield); ^{1}H NMR (500 MHz, CD_2Cl_2) δ 8.31 (d, *J* = 8.6 Hz, 2H), 7.69 (d, *J* = 8.8 Hz, 2H), 7.67 (d, *J* = 8.3 Hz, 2H), 7.29 (t, *J* = 7.2 Hz, 1H), 7.25 – 7.18 (m, 3H), 7.02 (s, 1H), 6.93 (d, *J* = 4.0 Hz, 1H), 6.91 (d, *J* = 5.4 Hz, 1H), 6.83 (d, *J* = 5.0 Hz, 1H), 6.40 (d, *J* = 4.7 Hz, 2H), 6.37 (d, *J* = 3.8 Hz, 1H), 4.59 (d, *J* = 6.6 Hz, 2H), 4.10 (q, *J* = 7.0 Hz, 2H), 2.39 (s, 3H), 1.44 (t, *J* = 6.9 Hz, 3H); ^{13}C NMR (126 MHz, CD_2Cl_2) δ 162.6, 157.5, 148.9, 148.4, 142.1, 138.4, 134.9, 134.0, 133.5, 132.0, 131.9, 130.2, 129.5, 129.5, 129.4, 129.3, 129.2, 125.4, 124.0, 121.0, 115.9, 112.7, 112.0, 64.5, 45.6, 21.6, 15.0; HRMS (ESI+) calcd. for $C_{31}H_{27}BF_2N_4O_3$, $[M+Na]^+$: 575.2042, found: 575.2041.

C. NMR Spectra

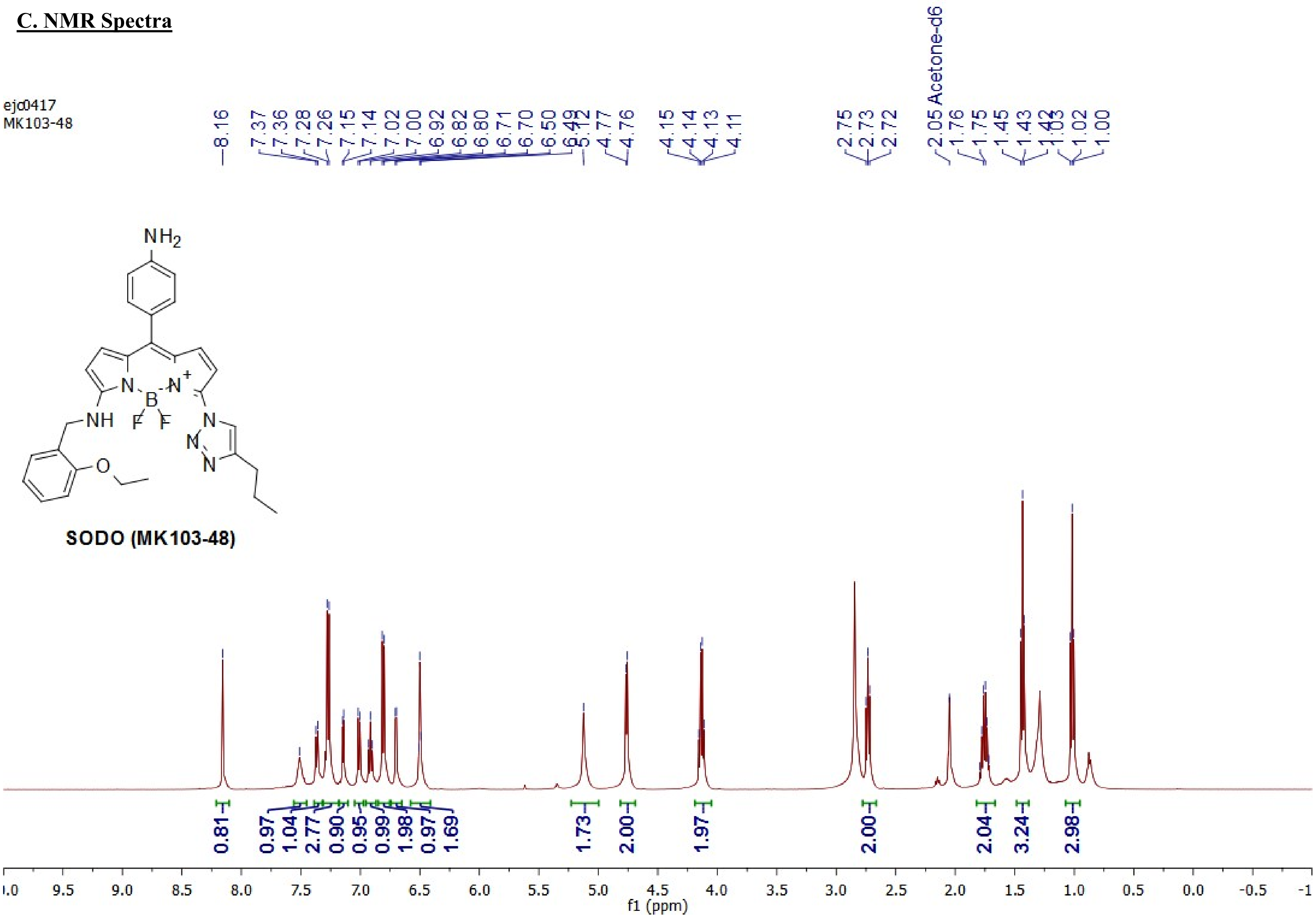

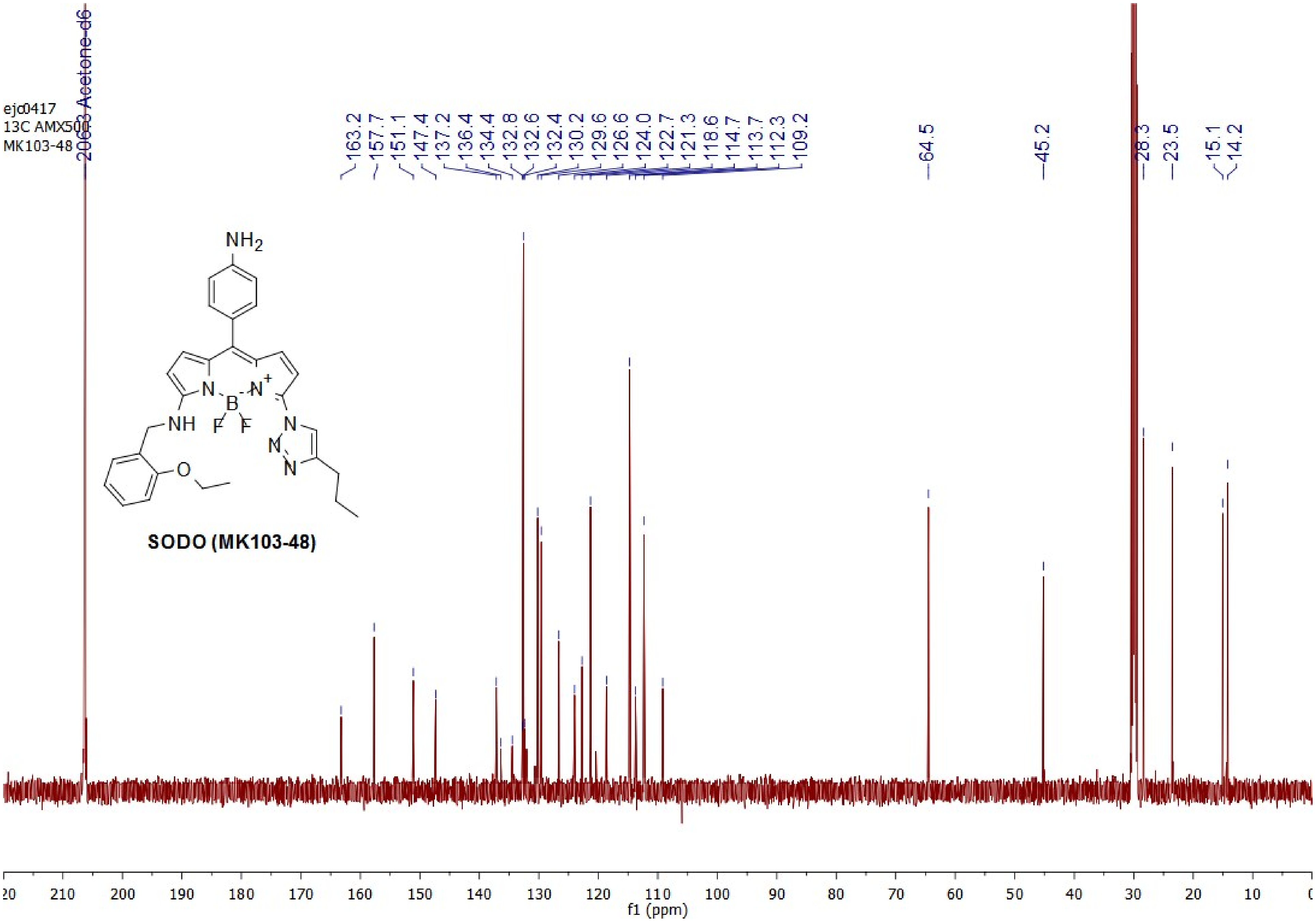

SODO (MK103-48)

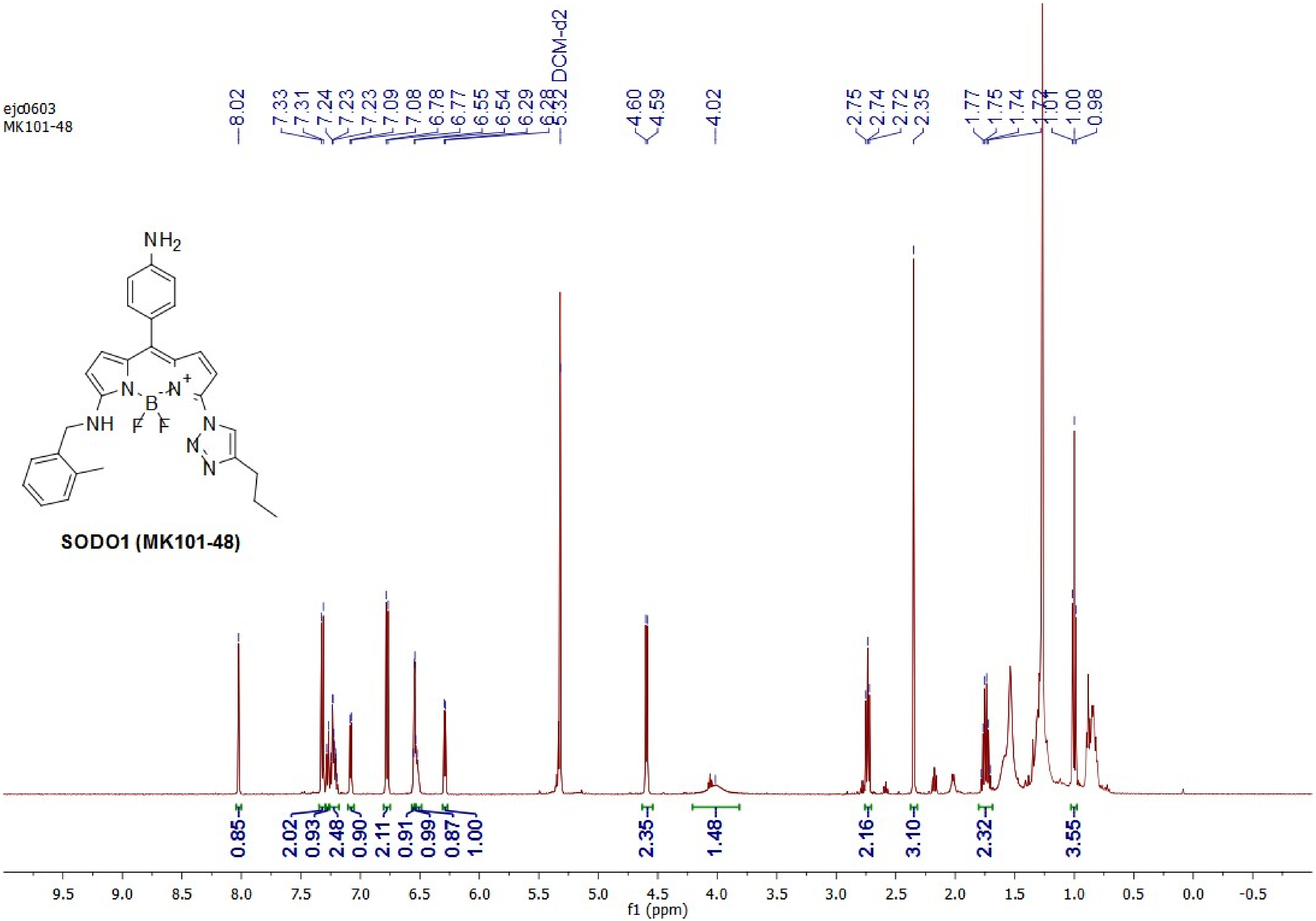

ejd0603
MK101-48
8.02
7.33
7.31
7.24
7.23
7.23
7.09
7.08
6.78
6.77
6.55
6.54
6.29
6.28
5.32 DCM-d2
4.60
4.59
4.02
2.75
2.74
2.72
2.35
1.77
1.75
1.74
1.72
1.01
1.00
0.98
NH2
N
B
N+
NH
F
F
N
N
N
SODO1 (MK101-48)
0.85
2.02
0.93
2.48
0.90
2.11
0.91
0.99
0.87
1.00
2.35
1.48
2.16
3.10
2.32
3.55
9.5
9.0
8.5
8.0
7.5
7.0
6.5
6.0
5.5
5.0
4.5
4.0
3.5
3.0
2.5
2.0
1.5
1.0
0.5
0.0
-0.5
f1 (ppm)

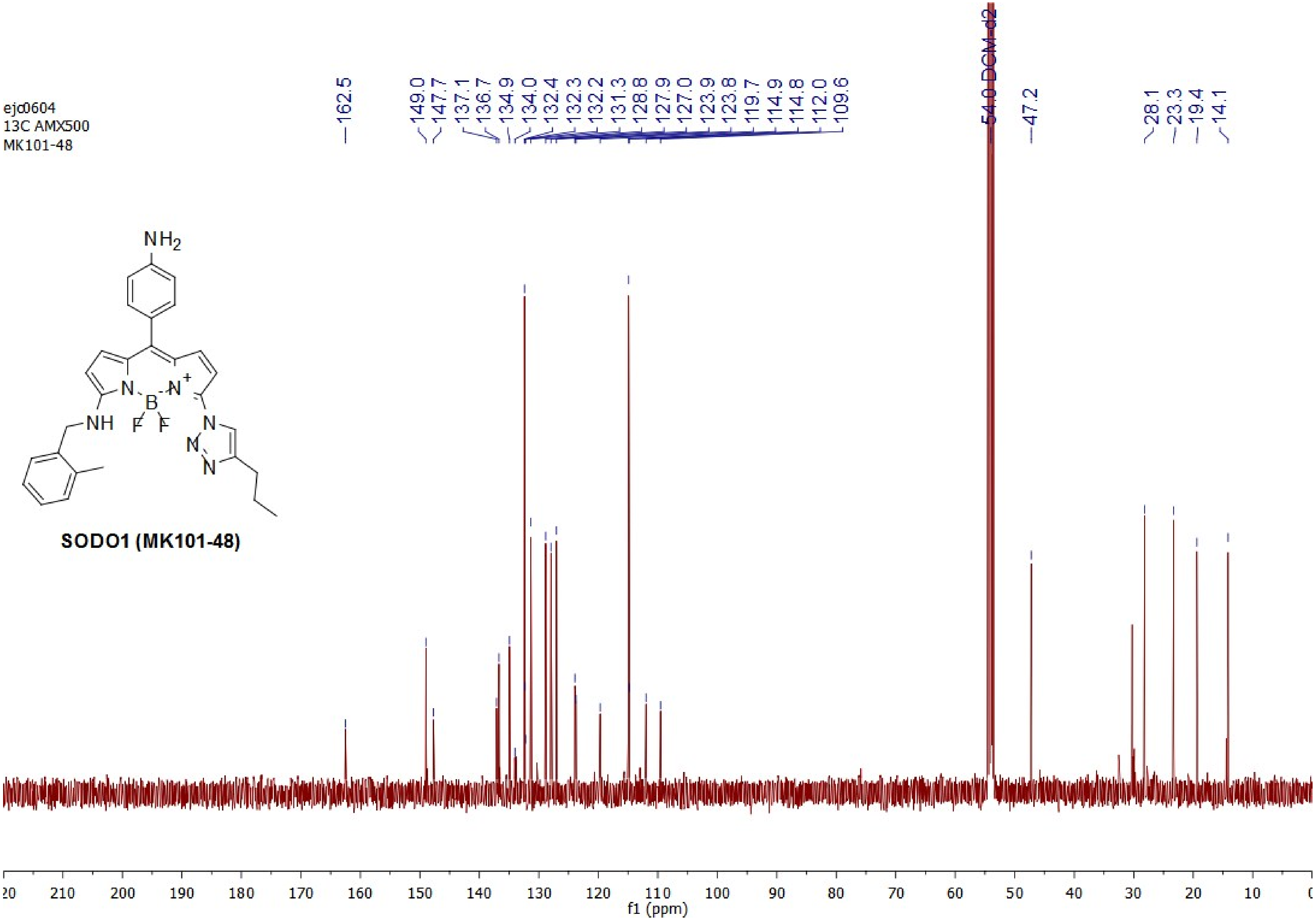
ejd0604
13C AMX500
MK101-48
162.5
149.0
147.7
137.1
136.7
134.9
134.0
132.4
132.3
132.2
131.3
128.8
127.9
127.0
123.9
123.8
119.7
114.9
114.8
112.0
109.6
54.0 DCM-d2
47.2
28.1
23.3
19.4
14.1
SODO1 (MK101-48)
f1 (ppm)

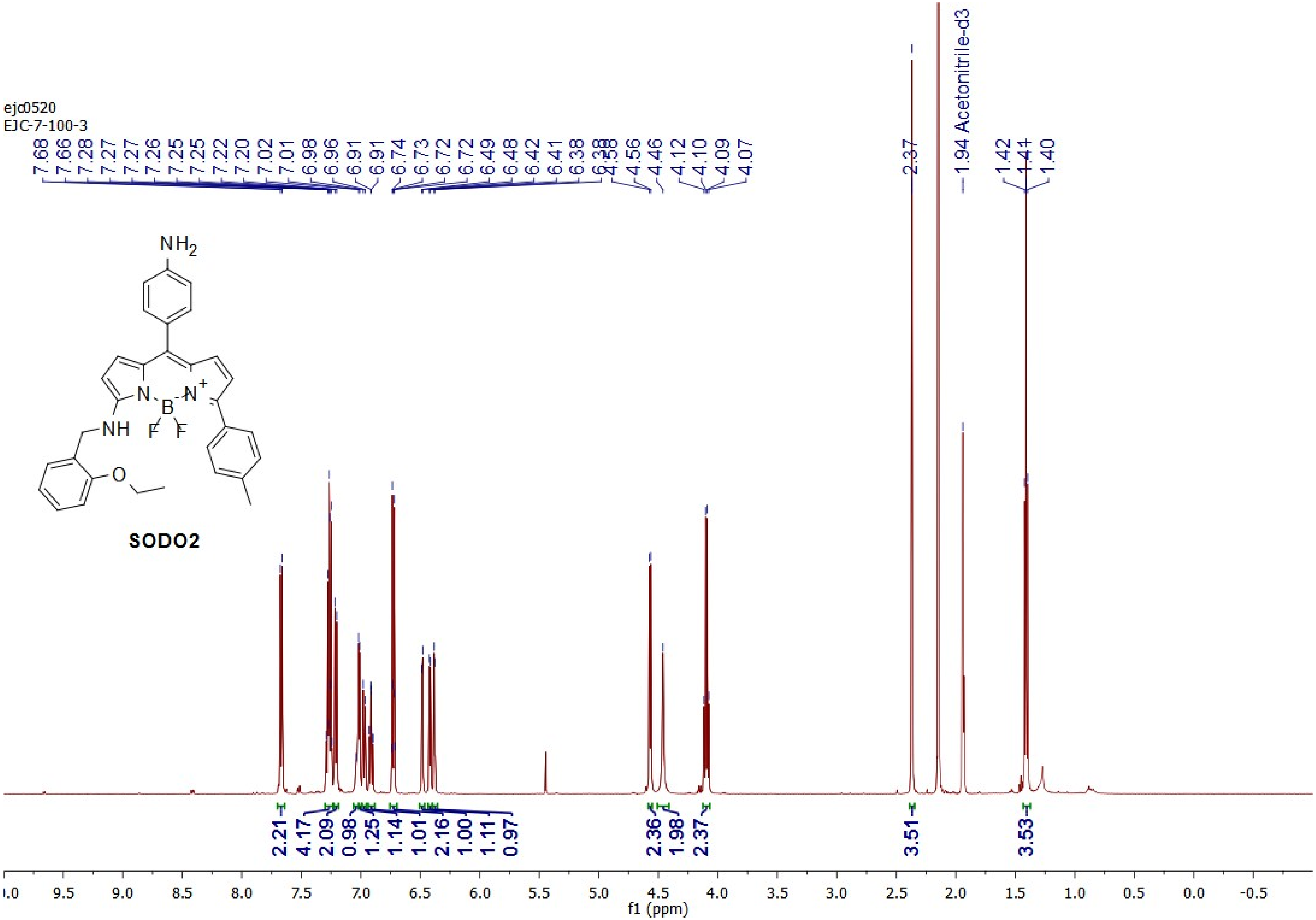
ejc0520
EJC-7-100-3
7.68 7.66 7.28 7.27 7.27 7.26 7.25 7.25 7.22 7.20 7.02 7.01 6.98 6.96 6.91 6.91 6.74 6.73 6.72 6.72 6.49 6.48 6.42 6.41 6.38
4.56 4.46 4.12 4.10 4.09 4.07
2.37
1.94 Acetonitrile-d3
1.42 1.41 1.40
NH_2
N
B
N^+
NH
F F
O
SODO2
2.21 4.17 2.09 0.98 1.25 1.14 1.01 2.16 1.00 1.11 0.97
2.36 1.98 2.37
3.51
3.53
f1 (ppm)

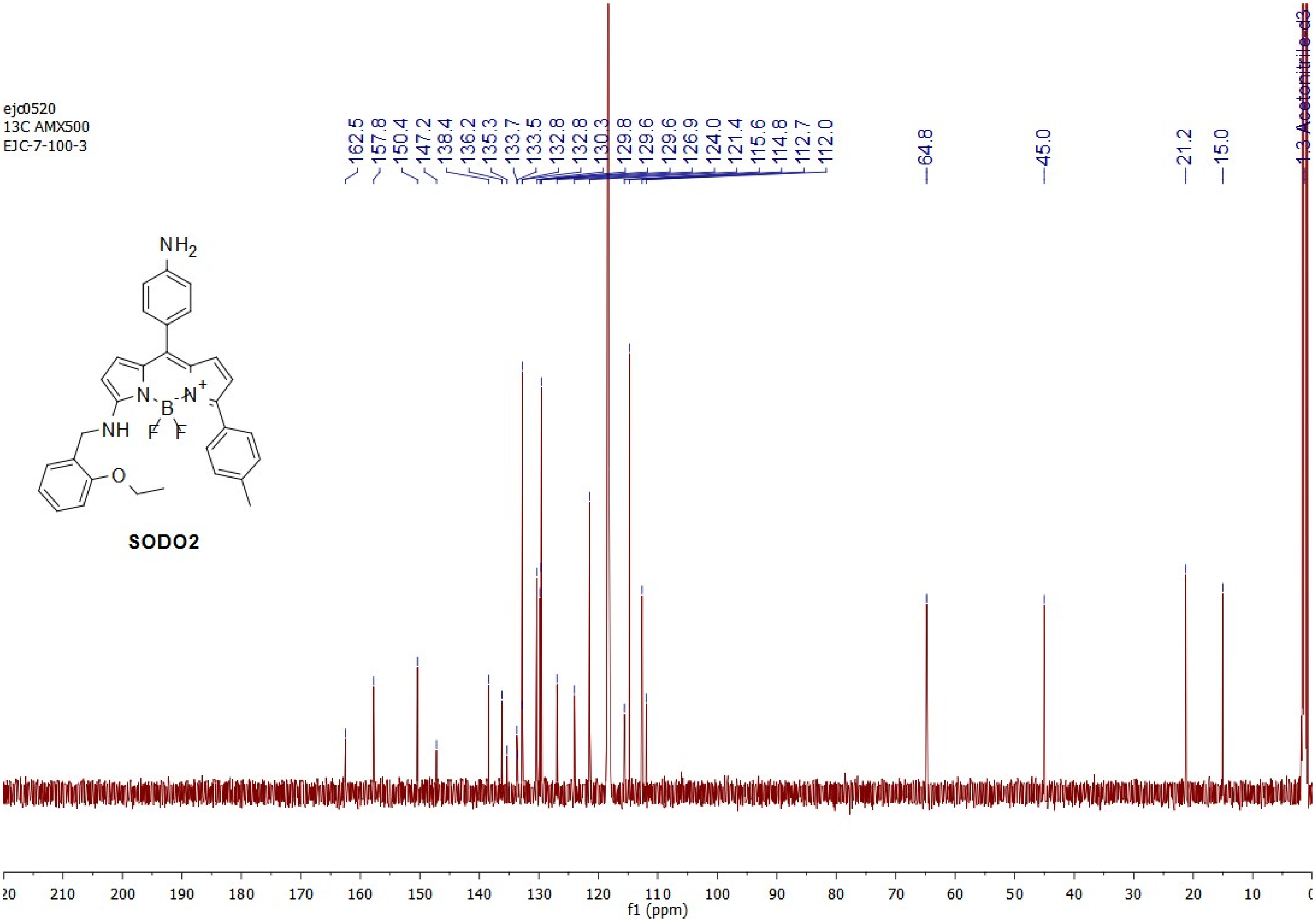
ejc0520
13C AMX500
EJC-7-100-3
162.5
157.8
150.4
147.2
138.4
136.2
135.3
133.7
133.5
132.8
132.8
130.3
129.8
129.6
129.6
126.9
124.0
121.4
115.6
114.8
112.7
112.0
64.8
45.0
21.2
15.0
1.3 Acetonitrile-d3
NH2
N
B
N+
NH
F
F
O
SODO2
210
200
190
180
170
160
150
140
130
120
110
100
90
80
70
60
50
40
30
20
10
f1 (ppm)

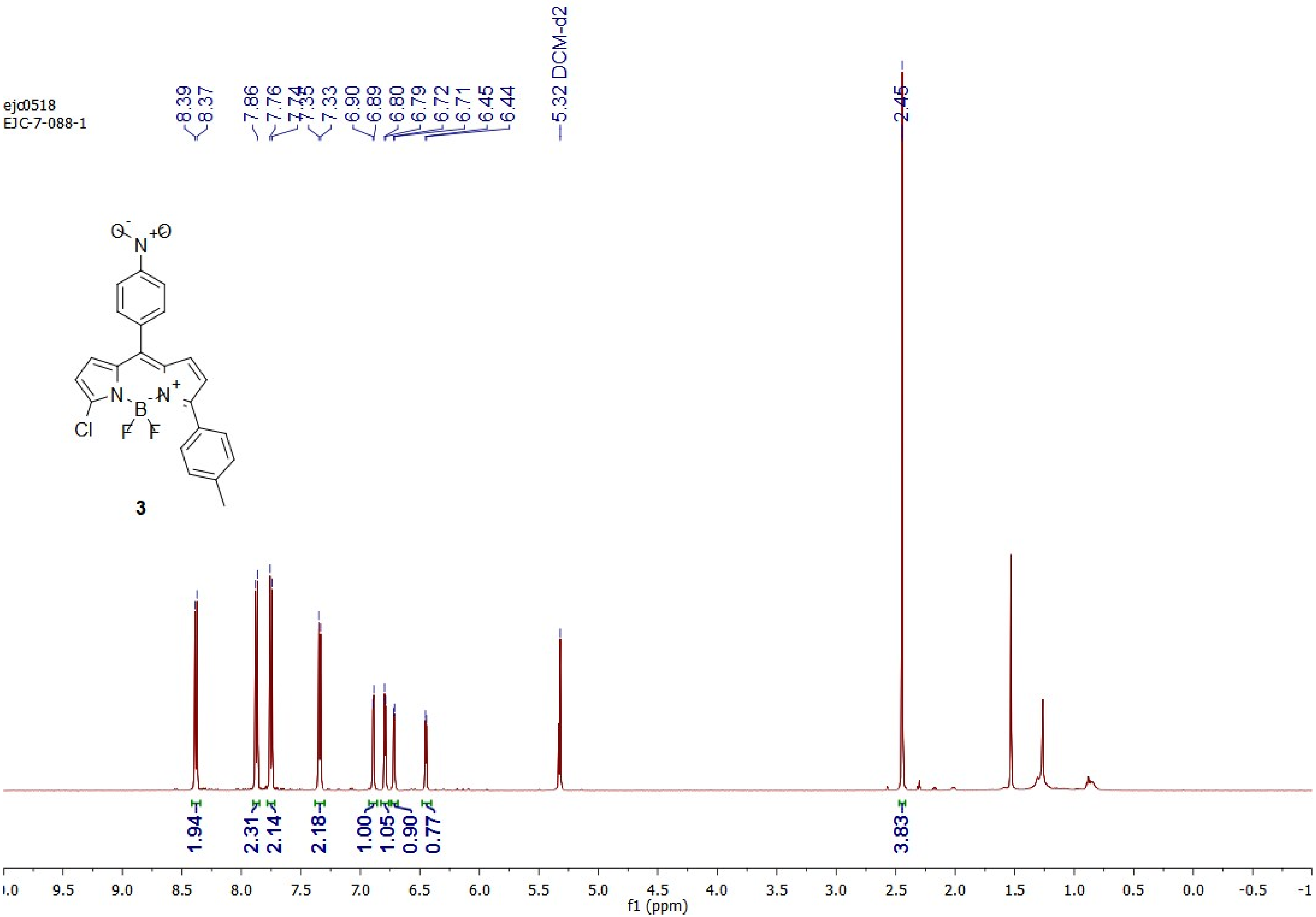
ejc0518
EJC-7-088-1
8.39
8.37
7.86
7.76
7.74
7.35
7.33
6.90
6.89
6.80
6.79
6.72
6.71
6.45
6.44
5.32 DCM-d2
2.45
N
O
O
N
B
N
Cl
F
F
3
1.94
2.31
2.14
2.18
1.00
1.05
0.90
0.77
3.83
9.5
9.0
8.5
8.0
7.5
7.0
6.5
6.0
5.5
5.0
4.5
4.0
3.5
3.0
2.5
2.0
1.5
1.0
0.5
0.0
-0.5
f1 (ppm)

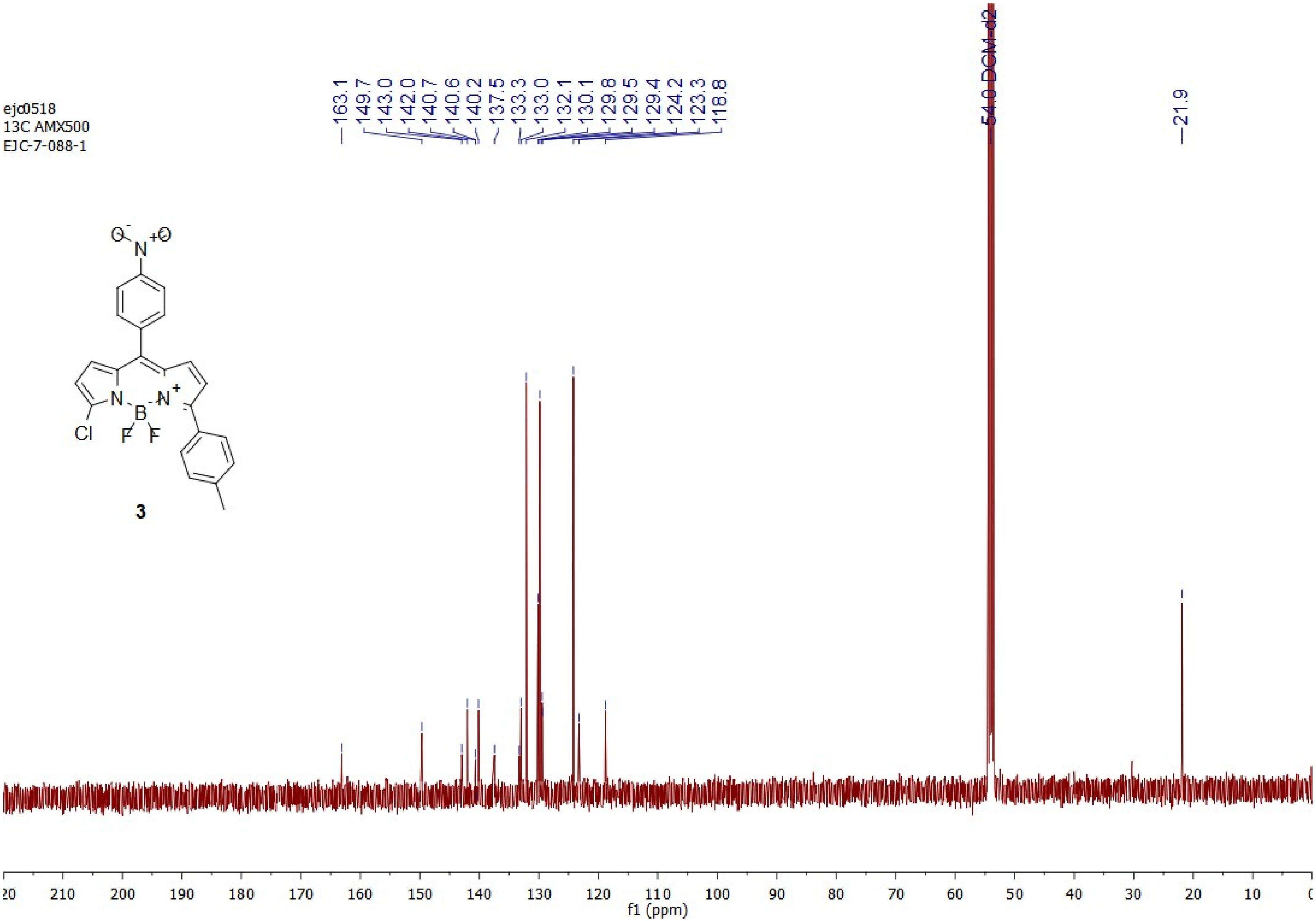
ejc0518
13C AMX500
EJC-7-088-1
163.1
149.7
143.0
142.0
140.7
140.6
140.2
137.5
133.3
133.0
132.1
130.1
129.8
129.5
129.4
124.2
123.3
118.8
54.0 DCM-d2
21.9
3
f1 (ppm)

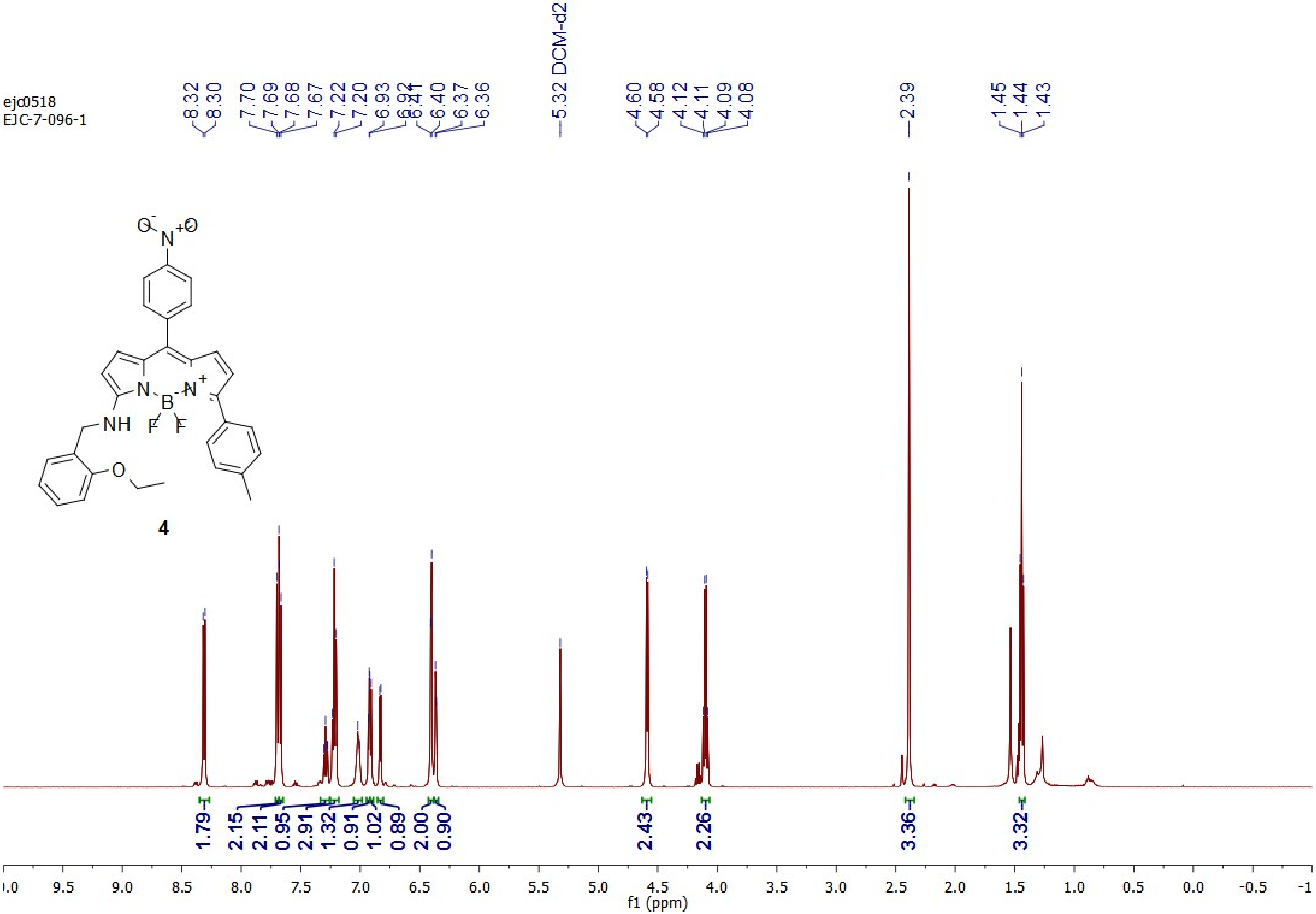
ejc0518
EJC-7-096-1
8.32 8.30 7.70 7.69 7.68 7.67 7.22 7.20 6.93 6.92 6.41 6.40 6.37 6.36
5.32 DCM-d2
4.60 4.58 4.12 4.11 4.09 4.08
2.39
1.45 1.44 1.43
NH
N
B
F F
O
4
1.79 2.15 2.11 0.95 2.91 1.32 0.91 1.02 0.89 2.00 0.90
2.43 2.26 3.36 3.32
9.5 9.0 8.5 8.0 7.5 7.0 6.5 6.0 5.5 5.0 4.5 4.0 3.5 3.0 2.5 2.0 1.5 1.0 0.5 0.0 -0.5
f1 (ppm)

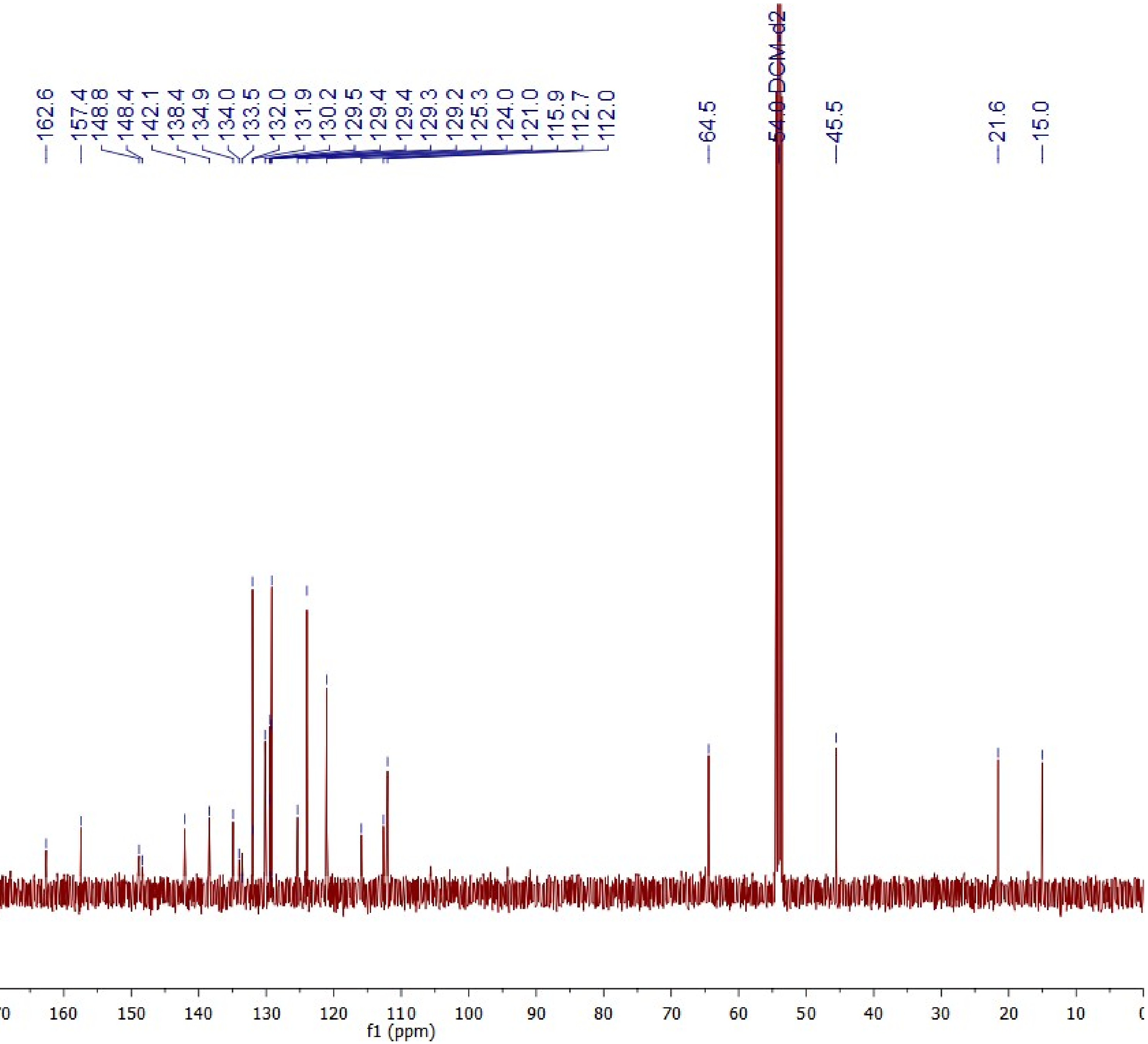
ejd0518
13C AMX500
EJC-7-096-1
162.6
157.4
148.8
148.4
142.1
138.4
134.9
134.0
133.5
132.0
131.9
130.2
129.5
129.4
129.4
129.3
129.2
125.3
124.0
121.0
115.9
112.7
112.0
64.5
54.0 DCM-d2
45.5
21.6
15.0
210 200 190 180 170 160 150 140 130 120 110 100 90 80 70 60 50 40 30 20 10
f1 (ppm)

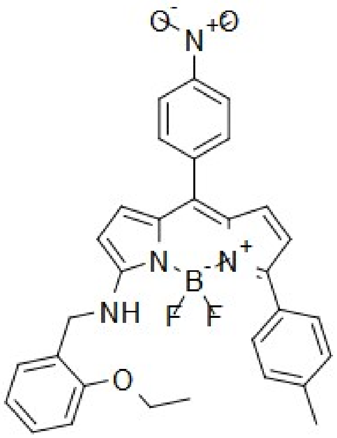
O
N
O
N
B
N
NH
F
F
O

4

D. References

1. J. H. Chen, K. Stoeber, S. Kingsbury, S. E. Ozanne, G. H. Williams and C. N. Hales, *J. Biol. Chem.*, 2004, **279**, 49439-49446.

2. E. Fei, N. Jia, M. Yan, Z. Ying, Q. Sun, H. Wang, T. Zhang, X. Ma, H. Ding, X. Yao, Y. Shi and G. Wang, *Biochem. Biophys. Res. Commun.*, 2006, **347**, 406-412.

3. Y. Ohara, T. E. Peterson, H. S. Sayegh, R. R. Subramanian, J. N. Wilcox and D. G. Harrison, *Circulation*, 1995, **92**, 898-903.

4. R. W. Strange, S. Antonyuk, M. A. Hough, P. A. Doucette, J. A. Rodriguez, P. J. Hart, L. J. Hayward, J. S. Valentine and S. S. Hasnain, *J. Mol. Biol.,* 2003, **328**, 877-891.

5. J. M. Word, S. C. Lovell, J. S. Richardson and D. C. Richardson, *J. Mol. Biol.*, 1999, **285**, 1735-1747.

6. O. Trott and A. J. Olson, *J. Comput. Chem.*, 2010, **31**, 455-461.

7. J. C. Er, M. K. Tang, C. G. Chia, H. Liew, M. Vendrell and Y.-T. Chang, *Chem. Sci.*, 2013, **4**, 2168-2176.
